# Determinants of food security among people from refugee backgrounds resettled in high-income countries: A systematic review and thematic synthesis

**DOI:** 10.1371/journal.pone.0268830

**Published:** 2022-06-02

**Authors:** Tina Gingell, Kate Murray, Ignacio Correa-Velez, Danielle Gallegos

**Affiliations:** 1 School of Nutrition and Exercise Sciences, Queensland University of Technology (QUT), and Woolworths Centre for Childhood Nutrition Research (WCCNR), South Brisbane, QLD, Australia; 2 School of School of Psychology and Counselling, QUT, Kelvin Grove, QLD, Australia; 3 School of Public Health and Social Work, QUT, Kelvin Grove, QLD, Australia; 4 School of Nutrition and Exercise Sciences, QUT, and WCCNR, South Brisbane, QLD, Australia; McGill University, CANADA

## Abstract

Food is intrinsically linked to culture, identity, and for people with lived refugee experiences, cultural foods are a critical part of settlement into a new country, which is often a time of high stress and dislocation from friends and family. However, cultural foods in settlement countries may be unavailable or inaccessible, adversely impacting on food security. This systematic review aimed to identify facilitators and barriers to accessing foods in high-income countries for people with lived refugee experiences. Sixteen health databases were searched from June 2020 and April 2021 and 22 articles met the inclusion criteria. Bias was assessed using a modified thematic synthesis method and the relevant Joanna Briggs Institute risk assessment checklist. Findings were thematically synthesised and the socio-ecological model and postcolonialism were used as a lens through which the data was viewed. Analysis revealed three themes: “Practicalities and Pragmatism”; “Identity, Belonging and Placemaking”; and “Postcolonial and Societal Influences”. The determinants of food security were present across all levels of the socio-ecological model and people with lived refugee experiences used practical and pragmatic strategies to feed their families. Food was intrinsically linked to identity, belonging and placemaking, and as such, people preferred consuming cultural foods. Societies adversely affected the food security of people from refugee backgrounds by limiting their access to resources and restricting cultural food gathering practices, impacting on their ability to access or afford foods, especially cultural foods. To improve food security for people with lived refugee backgrounds, governments and organisations should collaborate with the cultural communities with lived experiences of accessing cultural foods, appreciate their strengths, and recognise the value of social and cultural capital.

## Introduction

For people with lived refugee experiences, the decision to leave their home is generally involuntary, may be unexpected and sudden, and can be a time of high stress and dislocation from family and support networks [1, 2]. On arrival in the destination country, there is a shift from resettlement, the process of seeking asylum and being granted protection by the host country, to settlement, where people move towards a sense of belonging [3]. Eating habits are driven by food availability, cultural and religious practices, societal norms, individual experiences, and taste preferences, and as such, food is intrinsically linked to identity and the maintenance of culture [4–7]. Food is essential to the experience of belonging, placemaking and building connections with community [3]. Thus, food is a critical element in the settlement process, and when there are difficulties accessing culturally appropriate foods, termed hereafter cultural foods, settlement may be significantly disrupted, and this disruption can extend to household food security (HFS).

Food Security (FS) exists when “all people at all times have physical, *social*, and economic access to sufficient, safe and nutritious food to meet their dietary needs and *food preferences* for an active and healthy life” (our emphasis) [8 p7]. The important role of cultural foods when establishing FS is implied by the terms “social” and “food preferences” included in the definition. In recent years, access to cultural foods has increasingly been acknowledged as a driver of FS [4]. Food insecurity (FI) is when one or more of the conditions of FS is absent. This suggests that FI exists when cultural foods are not available, cannot be accessed, or are unable to be utilised. FI has been associated with negative health outcomes, such as hypertension, hyperlipidaemia, heart disease, overweight and obesity (in women), and poor mental health [9–14].

For migrants from refugee backgrounds, there are additional accumulative stressors during pre-flight, flight, exile, resettlement and settlement periods [2] which may create further unique challenges for maintaining FS. Despite the challenges imposed by experiences of persecution and forced migration, people with lived refugee experiences display resilience and fortitude in feeding their families [6, 15]. There is evidence to suggest there may be a rapid adaption to the food environment [6, 16], the development of strong social support networks enhancing food accessibility [16, 17], and communities exerting influence over the food environment through supply of previously unavailable culturally specific foods within the new environment [6, 18].

The term “refugee” can be used to frame an individual as vulnerable, traumatised, and reliant on a system of aid with no agency to improve their circumstances [3]. The label does not necessarily encompass other aspects of a person’s life preceding or following the experience of persecution and forced migration [3, 18]. Consequently, to recognise the varied life experiences, this review uses the term “with lived refugee experiences” or “from refugee backgrounds” to highlight that the refugee experience is only one aspect of a person’s life journey.

Due to the increasing numbers of people with lived refugee experiences in high-income countries [19] there is growing interest in the unique determinants of FS for people from refugee backgrounds. Three systematic reviews recently reviewed this topic [17, 20, 21]. Lawlis and Islam [17] investigated FS by examining the challenges to achieving the four dimensions of FS for people resettled in Australia. Mansour, Liamputtong and Arora [20] explored the determinants of FI in high-income countries of people who migrated from the Middle East and North Africa. In both, a small number of articles were selected for review (seven [17] and three [20], respectively). Additionally, Wood et al. [21] undertook a scoping review to identify factors affecting FS of people with lived refugee experiences in high-income countries. However, they did not state a methodology or theoretical framework underpinning the review. The current systematic review is unique as it includes all people with lived refugee experiences, regardless of country of origin, and broadly covers all high-income resettlement countries. It uses a thematic synthesis methodology underpinned by the Socio-Ecological Model (SEM) and postcolonialism frameworks to generate a deeper understanding of the determinants of food insecurity at the household level (household food insecurity (HFI)), for people from refugee backgrounds living in high-income country contexts.

The SEM is a way to frame problems while considering the influences of social contexts and systems [22]. It has previously been used in literature reviews as a lens to explore influences on dietary behaviours [23–25]. The SEM allows FS to be viewed as a complex system which is influenced by individual, interpersonal, institutional, community/environment, and public policy factors [24]. When the interaction between the factors is fully explored, effective interventions can be developed to target the multifaceted determinants of FI. Additionally, postcolonial theory is used as another lens to explore how colonisation has infiltrated social and political structures, marginalising other ways of knowing [26, 27]. Postcolonial theory is relevant as people from refugee backgrounds are largely originating from countries previously colonised, and resettling in countries that were colonisers or emerged as nation states from colonial invasion or Indigenous lands. Using this framing encourages critical examination of how historical and ideological functioning in a country affect the FS of people from different cultures.

This review aims to identify the challenges people with lived refugee experiences face when accessing food and uses a strengths-based approach to understand how these challenges have been overcome to inform the creation of more effective strategies.

## Methodology and methods

### Methodology

This review has been undertaken using thematic synthesis methodology [28]. Thematic synthesis draws on the concepts of a qualitative research approach called thematic analysis. It describes a way of analysing the findings of multiple studies to generate new themes which go beyond the findings of the original studies. The review has been conducted in accordance with the preferred reporting items for systematic review (PRISMA) [29].

The review team included members with extensive experience working with people with lived refugee experiences and food security among diverse populations.

### Study protocol registration

The protocol of this systematic review was registered with Prospero (Registration ID: CRD42020190971).

### Search strategy

Electronic databases searched included CINAHL, Consumer Health Databases, Health & Medical Collection, Healthcare Administration Database, Nursing & Allied Health Database, Public Health Database, Social Sciences Database, Web of Science, PubMed, Embase, Scopus, AMI, APAIS-Health, Health & Society, Health Collection, and RURAL. Search terms included a combination of “refugee”, “food security” and “food insecurity” and their synonyms. Examples of search terms can be found in S1 Table. Searches were originally conducted in June 2020 for articles published between 1990 and 2020 in English. In April 2021, searches were rerun for articles published between 2020 and 2021 in English and these were added to the original results.

Inclusion criteria: at least 50% of participants were people with lived refugee experiences of any age; participants had been resettled into the community of a high-income country providing a resettlement program registered with UNHCR. Identification as a high-income country was as per World Bank criteria [30]; peer reviewed primary evidence (qualitative, quantitative, mixed methods); and published in English between 1990 and March 2021. Exclusion criteria included: articles where the primary focus did not include factors or determinants contributing/inhibiting individual or household food security (FS) or its dimensions; papers which described eating patterns or lifestyle choices, health education needs and those that focused on prevalence without describing the determinants; and papers where the focus was on people seeking asylum. Settlement laws and transition agreements are different for people seeking asylum to those with legal refugee status, and they may not be resettled in the community. Therefore, the two groups face different and unique challenges to FS, which falls outside the scope of this review.

Using Rayyan QCRI [31], two of the authors (TG,DG) independently screened titles and abstracts against eligibility criteria. The full text of all articles was reviewed independently by the same authors with reasons for exclusion noted. Conflicts unable to be resolved were mediated by a third author (KM). All selected articles for full text review had forwards and backwards citation searching to identify other relevant articles for inclusion. Forwards searching involved using the “Times Cited” functionality in the Web of Science database, or where not listed, Google Scholar “Cited by” functionality. Backwards searching involved hand searching the reference list of selected articles. All articles identified during forwards and backwards citation searching underwent the same process as detailed for the original search.

### Assessment of methodological rigor

Qualitative articles were critically appraised using a modified version of the quality assessment tool by Thomas and Harden [28], incorporating aspects of the Critical Appraisals Skills Programme (CASP) systematic reviews checklist [32], and results were summarised as “good”, “fair” or “poor” in each quality category. Quantitative articles were critically appraised using the Joanna Briggs Institute (JBI) critical appraisal checklist for analytical cross-sectional studies [33] and the overall quality was ranked as “good”, “fair” or “poor”. Mixed methods articles were assessed by both the JBI critical appraisal checklist for case studies and the modified Thomas and Harden quality assessment tool [28, 32, 33].

### Data extraction

Data extraction forms were piloted on the first two articles reviewed (representing ten percent) and independently piloted by a second author (KM) to ensure all relevant information was captured. It was not necessary to contact any individual authors of articles for additional information. Data collected from the studies included the location and dates of data collection in the article, study design, methodology, community consultation undertaken during the study, participant details, recruitment methods, data collection tools, data collection method, translation method, data analysis method and relevant findings. In the case of quantitative studies, findings included the principal summary measures of hazard ratio, relative risk, and odds ratios. For qualitative studies, findings were deemed to be all text in the results and discussion sections of the articles under review.

### Data analysis

All selected studies were eligible for synthesis. Data was initially analysed using thematic synthesis to inductively identify emerging themes, and then the SEM and postcolonialism was deductively applied as a lens through which the data was viewed [22, 28]. Coding was conducted by the lead author and ten percent of articles were independently coded by a second author (DG) and compared. A code book was created inductively, which was iteratively refined throughout data analysis. In brief, the following data analysis steps were undertaken. *Stages one and two—coding text and developing descriptive themes*: Line-by-line coding occurred for all findings which related to the research question. The text for each code was reviewed by the lead author, summarised and a graphic representation developed which was reviewed independently by the three other authors. All four authors collaboratively discussed each code and iteratively reviewed the categories to develop descriptive themes. *Stage three—generating analytical themes*: all authors collaboratively discussed the inferred barriers and facilitators from the perspectives of the people with lived refugee experiences captured by the descriptive themes to develop analytical themes and a graphic representation of these themes. Analytical themes and the graphical representation were discussed and checked for authenticity and verification with people working closely with, or who themselves had, lived refugee experiences (n = 3), residing in Southeast Queensland, Australia, during one-on-one meetings that lasted approximately one hour.

## Results

**Fig 1:**
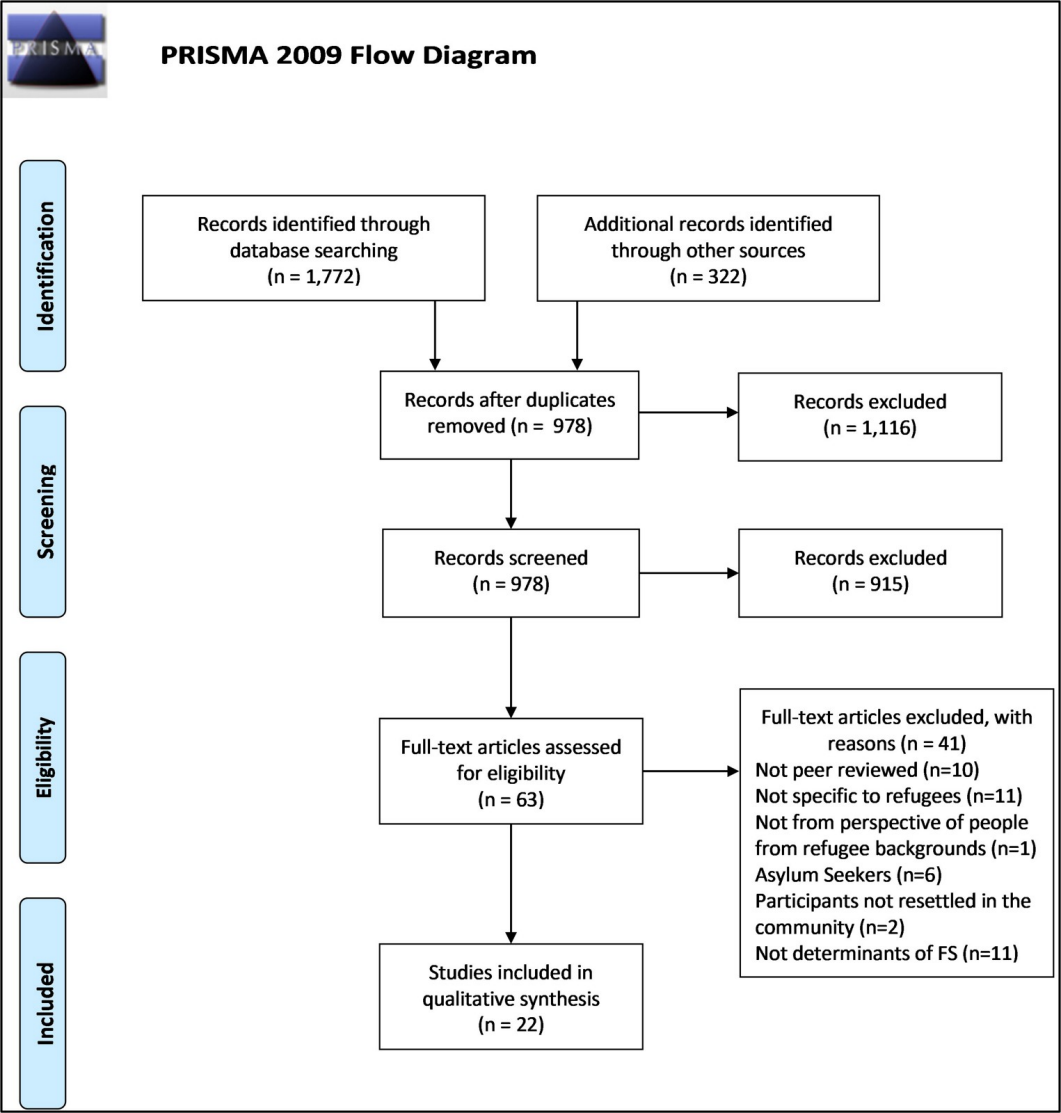
PRISMA 2009 flow diagram of article selection.

Twenty-two articles were selected for data extraction. Fig 1 provides the PRISMA diagram of the selection process. Data was extracted from the full text of 22 articles and the quality of these articles were assessed. No articles were excluded based on their quality assessment, which can be found in S2 Table. Table 1 provides the characteristics of the articles included in this review and further detail is provided in S3 Table.

**Table 1:** Summary details of selected articles (n = 22) included in the systematic review and synthesis.

Author Location	Data collection dates Methodology Method	Sample size and characteristics	Ethnicity; Immigration status	Recruitment Data collected	
Quantitative Studies (n = 7)					
Anderson et al. [34] Atlanta, USA	Jul-Oct 2002 Interviewer-administered individual interviews	N = 49 **Sex**: not stated **Time since resettlement**: 29% 0-2yrs (n = 14), 61% 2–4 yrs (n = 30), 10.2% 4-5yrs (n = 5). **Inclusion/Exclusions**: Self-identified caregiver with 1. ≥ one child under 3; 2. a legally resettled Sudanese parent in the USA; 3. parent legally resettled USA resident for < 5 yrs.	Sudanese; Not stated	**Recruitment**: Purposive from voluntary resettlement agencies, churches and other community group meetings and snowball sampling. Self-identified caregiver in each household interviewed during home visit conducted in Arabic, Nuer, or Dinka by bi-lingual interviewers from refugee outreach organisations of Sudanese origin. **Data collected**: Demographic indicators for each index child and caregiver; household, health and budget; diet and shopping; household food security; baby feeding and birth; and social life.	
Dharod et al. [35] Maine, USA	Oct 2006-Dec 2007 Interviewer-administered individual interviews	N = 180 **Sex:** 100% female **Time since resettlement**: 56% 1–3 yrs (n = 109), 22% 4–6 yrs (n = 43), 22% ≥ 7 yrs (n = 43). **Inclusion/Exclusions**: 1. Somali women residing in Lewiston area; 2. main meal preparers of the household; 3. Mother of ≥ 1 child 2 to 12 yrs old	Somali; Not stated	Recruitment: Not stated. Conducted at participants home in preferred language by trained bilingual Somali women facilitators **Data collected**: FI, sociodemographic, dietary habits, anthropometric measurements.	
Gallegos et al. [36] Perth, Australia.	2002 to 2003 (9mths) Interviewer-administered individual interviews	N = 51 **Sex**: not stated Time since resettlement: <12 mths Inclusion/Exclusions: None	Afghanistan (n = 8), Middle East/Iran/ Iraq (n = 15), West Africa (n = 3), Horn of Africa (n = 6), North-East Africa (n = 9), Former Yugoslavia (n = 10); 67% humanitarian entrants, 28% temporary protection visas, 6% not stated classification	**Recruitment:** Convenience sampling (selected by early intervention team (EIT)). EIT caseworkers with assistance from accredited interpreters EIT given discretion to choose when/where to conduct the questionnaire. Some for every new client others after establishing relationship with client. Instruction sheet provided to EIT workers. EIT workers asked not to prompt for this question. Not stated what language delivered. **Data collected**: Socio-demographic details, presence of FI, possible reasons for running out of food and any other comments regarding the issue.	
Gichunge et al. [37] South-East Queensland, Australia	Apr-Dec 2012 Interviewer-administered individual interviews	N = 71 households (383 household members). **Sex:** 89% female Time since resettlement: Avg 4.9yrs. **Inclusion/Exclusions**: Primary food preparers with: 1. A child under 18 yrs of age; primary food preparer speaks English or Swahili.	Burundi 75% (n = 53), Rwanda 13% (n = 9), and the Democratic Republic of Congo 13% (n = 9) (Africa Great Lakes region); Not stated	**Recruitment:** Purposive recruited from African churches, community meetings and settlement agencies then snowballing. Facilitator was bilingual researcher. Participants received AUD$25 grocery voucher **Data collected**: Demographic characteristics, household food security measurement, food frequency questionnaire.	
Hadley et al. [1] North-Eastern USA	Not stated Interviewer-administered individual interviews	N = 33 **Sex**: 100% female Time since resettlement: <5yrs. **Inclusion/Exclusions**: 1. Mother of Liberian origin and refugee or asylee status; 2. Living in the USA < 5 yeas; 3. Currently caring for a child < 5 yrs old. Effort was made by the interviewers to locate both employed and unemployed respondents	Liberian; Refugee or asylum status	**Recruitment:** Convenience sample recruited by word of mouth at the resettlement centre and in several community groups. Conducted privately at resettlement centre or at home by trained Liberian women well known in community. Participants provided US$10. Language not stated. **Data collected**: socio-demographics, migration history, social support, food security, dietary intake, shopping patterns, and acculturation.	
Hadley et al. [38] Mid-Western USA	2006 Interviewer-administered individual interviews	N = 281 **Sex**: 64% female Time since resettlement: Avg 48 mths **Inclusion/Exclusions**: 1. Having refugee status; 2. ≥ 18 yrs old	Sierra Leone, Liberia, Ghana, Somalia, Togo, and Meskhetian Turk (% not stated); Refugee or asylum status	**Recruitment**: Initially recruited through a local resettlement agency then snowballing. Conducted in participant’s language by trained interviewer of same gender and spoke same language. **Data collected**: Demographics, difficulty navigating the food-related environment, food security.	
Vu et al. [39] Atlanta, USA	Sept 2017-Apr 2018 Self-administered individual interviews	N = 162 **Sex**: 66% female Time since resettlement: Not stated. Inclusion/Exclusions: None stated.	51.9% (n = 84) Vietnamese, 16.0% (n = 26) Hispanic, 15.4% (n = 25) Burmese, 14.8% (n = 24) Bhutanese or Nepali, 1.2% (n = 2) Bengali, and 0.6% (n = 1) Cambodian; Not stated	**Recruitment**: Convenience sampling, two community health fairs and clients using services at partner organisations Survey in English and translated into Vietnamese, Spanish, Burmese, or Nepali. Fair participants were entered into a draw for US$50/$100 gift card, other participants provided US$5 incentive. **Data collected**: FI, acculturation, social connectedness, substance use (e.g., tobacco and alcohol), preventive care utilisation, vaccinations (e.g., hepatitis B vaccination and human papillomavirus vaccination), and other sociodemographic information.	
Qualitative Studies (n = 9)					
Burns et al. [40] Melbourne, Australia	Not stated Focus Groups	N = 33 **Sex:** 91% female Time since resettlement: 4mths to 3 yrs Other Inclusion/Exclusions: None.	Somali; Refugees	**Recruitment:** Snowballing through Somali workers and members of the Somali community. 4 gender specific focus groups held at 3 Somalian community locations facilitated by a Somali worker, nutritionist & project worker **Data collected (topics)**: Food beliefs and practices, issues relating to children’s food habits, food supply, food preparation and changes in food related issues that had occurred since arrival in Australia. Thematic analysis.	
Cordeiro et al. [41] Lowell, Massachusetts USA	Not stated Community based participatory research. Focus groups	N = 84 Sex: mixed Time since resettlement: Not stated. Other Inclusion/Exclusions: ≥ 15 yrs	Self-identified Cambodian (n = 49 + youths) & Brazilian (n = 16); Arrival in the USA as refugee/ immigrant or child of refugee/ immigrant	**Recruitment:** Convenience sampling (word of mouth and through local media programs, schools, community events, extension courses and community partners that serve low-income populations). 11 focus groups, including specific groups for adolescents, elderly, parents of children with disabilities, working parents, pregnant women, and ESOL learners. Adult groups held in Khmer or Portuguese with English-speaking moderators and community translators, youth groups held in English. **Data collected (topics)**: Personal and intergenerational experiences with food access and food security; safety net use; barriers to purchasing and consuming healthy food; and access to, and use of, community resources. Thematic analysis.	
Dharod et al. [42] Guilford County, North Carolina, USA	***Phase I*:** June 2010 to August 2011. ***Phase II*:** December 2011 to April 2012 Semi-structured interviews	Phase I N = 18. **Sex**: 100% female **Time since resettlement**: Avg 7 yrs. **Inclusion/Exclusions**: 1. Being a refugee mother of at least one child 12 yrs old or younger; 2. Being the main meal preparer for the household; 3. Currently living in Guilford County, North Carolina. Phase II N = 5 **Sex**: Mixed **Time since resettlement**: 6 yrs. **Inclusion/Exclusions**: 1. Held a medical degree; 2. Previously practiced medicine in their country of origin	**Phase I** Sudan, Liberia and Vietnam; Not stated **Phase II** Montagnard; Not stated	**Phase 1 Recruitment:** Snowballing (through personal contacts, networking or introduction by original participants) **Phase II Recruitment:** Recruited through community contacts developed through collaboration and building connections via community-engaged scholarship. ***Phase I*:** At participant’s home, Liberian in English and Sudanese in Arabic, Vietnamese in tribal language by bilingual female community outreach workers in presence of principal investigator. Participants received US$75 gift card. **Data collected**: socio demographics, what specific food item increased, lifestyle changes, and difference in food environment and dietary habits. ***Phase II*:**: At participant’s home or community site (eg Asian restaurant). Conducted in English. Participants received US$40 gift card. **Data collected**: Participants medical training history, lifestyle and food-related challenges that community members experienced in USA. Thematic analysis	
Hughes (43) Coffs Harbour, NSW, Australia	Not stated Ethnography and participatory research. Formal and informal interviews	N = 12–15 adult community members **Sex**: Mixed Time since resettlement: Not stated. Inclusion/Exclusions: None stated	Myanmar; Not stated	Recruitment Through connections made in ethnographic process. Interviews with individuals, families and small groups + participant observations in natural settings including homes, gardens and community events + documentary depicting several participants food journeys. Facilitated by Ethnographic researcher (no information provided). Data analysis not yet completed—this study represents initial data.	
Hughes (6) Coffs Harbour, NSW, Australia	10 mths (dates not specified) Focused ethnography with participatory methods. Community consultation sessions + walking interviews/home and garden tours + semi-structured and informal interviews with participants + participant observation	N = 12 main contributors **Sex**: Not stated Time since resettlement: Not stated. Inclusion/Exclusions: None stated.	Myanmar; Not stated	**Recruitment:** Purposeful and snowball sampling. Observations were recorded in a field journal and were filmed where appropriate consent was provided. Key findings were used to create a documentary film depicting the participants’ food journeys. Most interviews conducted in Burmese (common language) by ethnographic researcher (no information provided). Thematic analysis	
Judelsohn et al. [18] Buffalo, NY, USA	Not stated Participatory action research. Interviews + focus group	N = 28 **Sex**: Not stated Time since resettlement: Not stated Inclusion/Exclusions: Not stated	Burmese; Not stated	**Recruitment:** Snowballing recruited by community advisory group members and research team members (ties to Burmese community) through word of mouth and fliers. Interviews and focus groups conducted in preferred language by interpreter (Burmese, Karen or English). Facilitator: Not stated (see above). **Data collected**: Resettlement experience, especially pertaining to food and health experiences. Content and thematic analysis	
Kavian et al. [44] Adelaide, SA, Australia	May-Sept 2017 Grounded theory using social determinants of health (SDOH). Semi-structured interviews + field notes	N = 10 **Sex**: 100% female **Time since resettlement**: <2yrs. **Inclusion/Exclusions**: Afghani female refugees who lived in Australia for 2 yrs or less.	Afghani; Refugees (some on humanitarian vias and some sponsored by spouse	**Recruitment:** Snowballing using various strategies with numerous orgs working with refugees. Interviews conducted at convenient time and place in participant’s native language by the principal investigator who is bilingual in Farsi/Dari and English. **Data collected**: The social determinants of health in both the transition country and Australia. Iterative thematic analysis	
McElrone et al. [16] South-eastern USA	Dec 2017-Feb 2018 Socio-ecological model framework. Semi-structured interviews + field notes	N = 18 **Sex:** 100% female **Time since resettlement**: Avg 67 mths (5.6 yrs) **Inclusion/Exclusions**: 18 yrs of age or older, self-reported refugee status, and native of a Sub-Saharan African country.	67% Burundian (n = 12) and 33% Congolese (n = 6); Self-reported refugee	**Recruitment:** Word-of-mouth through refugee programs using network then snowballing. Conducted at participant’s preferred location in preferred language by principal Investigator with aid of interpreter. Participants were provided US$25 gift card. **Data collected**: Post resettlement FS, experiences regarding culturally familiar food access, food shopping, transportation to food outlets, meal preparation habits and cooking methods/equipment, and government nutrition assistance programs. Sociodemographic information. Thematic analysis using constant comparative method (grounded theory approach).	
Vatanparast et al. [45] Toronto & Sakatoon, Canada	Dec 2016-Feb 2017 Not stated. Semi-structured interviews	Phase 1			
		N = 54 **Sex**: 30% female **Time since resettlement**: <3 yrs. **Inclusion/Exclusions**: Resettled in two Canadian urban contexts, Toronto and Saskatoon, since November 2015	Syrian; All refugee status, either private, government or blended sponsorship	**Recruitment:** Non-probability snowball sampling and data saturation determined sample size. Contacts and connections with local refugee settlement agencies and community members were leveraged to recruit participants. Conducted at resettlement service agency locations; and community events in English or Arabic (based on preference) by trained bilingual interviewers (English and Arabic) **Data collected**: the challenges, barriers, cultural and gendered nature of FS and the adequacy of support services based on their experiences.	
		Phase II			
		N = 15 **Inclusion/Exclusions:** Key informants responsible for the implementation of refugee programming	As above	**Recruitment:** Range of settlement and community-based organisations (settlement officers/coordinators, policy developers, senior managers, program managers and directors), health-care professionals (dieticians and nurse practitioner) and government service employees (policy analyst and provincial program manager). Face-to-face interviews conducted at resettlement service agency locations and community events or via telephone, and in English (based on preference) by trained bilingual interviewers. **Data collected**: The capacity of service providers and agencies to support and respond to the FS issues facing Syrian refugees. Thematic analysis.	
Mixed Methods Studies (n = 6)					
Gichunge et al. [5] South- East Queensland, Australia	Quantitative				
	Apr 2012 Interviewer-administered questionnaire	N = 71 households **Sex:** 89% female **Time since resettlement**: Not stated. **Inclusion/Exclusions**: Primary food preparers from households with children under 18 yrs were recruited	Burundi 75%, Congolese and Rwandan; Not stated	**Recruitment:** Purposive recruited from African churches, community meetings and settlement agencies then snowballing. Interviews conducted in English or Swahili by bilingual researcher. Participants received AUD$25 grocery voucher. **Data collected**: Demographics and socioeconomic characteristics, food environment and household food inventory using a pre-determined list of household food inventory.	
	Qualitative				
	Apr 2013 Not stated Interviewer-administered individual interviews	N = 15 **Sex**: Not stated Time since resettlement: not stated Inclusion/Exclusions: As above.	Not stated; Not stated	**Recruitment:** Purposively selected from quantitative participants (no further details). Conducted in English or Swahili by bilingual researcher. **Data collected**: Where do you get the vegetables? Why do you have these vegetables in your home? What problems do you encounter when sourcing your traditional vegetables in your neighbourhood?	
Hadley et al. [13] USA	Quantitative				
	Not stated Interview administered survey	N = 101 **Sex**: Female. Time since resettlement: Avg 22.1 mths. **Inclusion/Exclusions**: 18 yrs or older, has a child under 5 yrs of age, living in the USA for less than 4 yrs, and claimed Liberia as country of birth	Liberian; Not stated	**Recruitment:** Service-based convenience and snowballing sampling. Recruited through primary resettlement agency, meeting points of the Women, Infants, and Children Program (WIC; a public assistance programme with set income criteria), church groups. Conducted in English (national language of Liberia) at participants home by West African women. **Data collected (baseline)**: Migration history, current household composition and economics, participation in food stamp programs, perceived difficulty with shopping and language. Data collected (6mth follow up): HFI in previous 6 mths	
	Qualitative				
	Not stated Ethnography Observations + conversations + informal interviews + formal interviews	N = 15 (formal interviews) **Sex**: Female. Time since resettlement: <4yrs. **Inclusion/exclusions**: 18 yrs or older, has a child under 5 yrs of age, living in the USA for less than 4 yrs, and claimed Liberia as country of birth	Liberian; Not stated	**Recruitment:** same as quantitative **Data Collected**: FI, dietary acculturation, food preparation and difficulties in the USA.	
Henderson et al. [46] Winnipeg, Canada (only qualitative relates)	Qualitative				
	Not stated Not stated. Photovoice + Interviewer-administered individual interviews + analytical memos	N = 12 (8 newcomers and 4 community workers) **Sex**: 2 men & 6 women (newcomers) Time since resettlement: 6mths - 6yrs. **Inclusion/Exclusions**: Length of time in Canada ≥ 6mths, a predominant role in food procurement and preparation in the household, ≥18 yrs old and interest in participating in a photovoice study. Newcomers who had a family member participating in the study were excluded. 4 community workers were recruited who: were involved with relevant programmes for North End community members, including nutrition education, cooking classes and gardening programmes.	Afghanistan, Bhutan, Burma, Congo, Iraq and the Philippines; 7 newcomers had refugee status (88%)	**Recruitment:** Purposively recruited through partner organisations, community workers and word of mouth. Participants provided with single use camera and instructed to take 15 photos of food environment. Interviews conducted in English with interpreters where required. Photographs bought to interview for discussion. Participants were paid CAD$25. **Data collected**: Photos of the food environment and experiences e.g. food purchases, food preparation and gardening activities. **Analytical memos**: Recorded throughout the study to evaluate effectiveness of interview questions, and document aspects of interview not captured in transcriptions.	
Judelsohn et al. [47] (only qualitative relates) Buffalo, NY, USA.	Qualitative				
	Not stated. Participatory action research. Interviewer-administered individual interviews	N = 28 **Sex**: 79% female 21% male. Time since resettlement: >6mths. **Inclusion/Exclusions**: Born in Burma, refugee status, lived in USA > 6mths, ≥ 18 yrs old.	Burmese; Refugee status		**Recruitment:** Snowballing recruited by CAG members and research team members (ties to Burmese community) through word of mouth and fliers. Conducted in preferred language, Burmese, Karen or English at preferred location by bilingual interviewers with ties to Burmese and Karen communities and accompanied by additional research team member. **Data collected**: How refugees navigate the food environment, challenges and how they overcome these.
Nunnery et al. [48] (only qualitative relates) County in South-eastern USA	Qualitative				
	March 2010 to November 2012. Not stated, analysed by pre-and post-resettlement factors. Secondary data analysis of 3 studies (Interviews collecting qualitative and quantitative data)	N = 97 **Sex**: Women. Time since resettlement: Avg 8yrs. **Inclusion/Exclusions**: 1. Came to the USA under refugee status or under the family reunification program; 2. 18 yrs of age or older; 3. The main meal preparer of their household; 4 Had children younger than 18 yrs of age.	Liberian (n = 33), Sudanese (n = 22) Montagnard’s (n = 42); Refugee status or family reunification	**Recruitment:** Snowball techniques such as networking, telephone invitations and referrals. Conducted in participants’ home. Liberian interviews in English, Sudanese and Montagnard in native language by bilingual community health workers of same ethnicity and living in the community of study group in presence of research team member. **Data collected**: Sociodemographic & FS, general experiences related to social and cultural changes during initial period of resettlement, current lifestyle, food shopping and dietary habits, pre-resettlement living conditions, health and food environment, and concerns and issues related to food and health in the USA.	
Peterman et al. [49] Lowell, Massachusetts, USA.	Quantitative				
	September to November 2007 and April to June 2008. In-person administered survey	N = 150 (data analysis) of 160 completed surveys of 196 households selected **Sex**: Female. **Time since resettlement**: Avg 19.3 yrs in USA. **Inclusion/Exclusions**: Cambodian women aged between 35 to 60 yrs Analysis conducted only on those that had been in the USA for ≥ 5 yrs.	Cambodian; Not stated	**Recruitment:** Random sample selected from 2007 Lowell City Census and telephone book; registered voters from University of Massachusetts Lowell; and clients from community agencies. Administered in person in preferred language of participant at participants house by trained survey administrators. **Data collected**: HFS, depression, acculturation, food stamp participation, and demographics.	
	Qualitative				
	April to May 2007. Not stated. Focus groups	N = 11 (2 focus groups) **Sex**: Female. Time since resettlement: Not stated. **Inclusion/Exclusions**: Women aged 30-65yrs.	Cambodian; Not stated	**Recruitment:** Recruited from clients of CMAA. Conducted in Cambodian by English speaking moderator, no other details provided. **Data collected**: Participants’ experiences with food on arrival in the USA and current access to food.	

Avg = Average, Yrs = Years, Mths = Months, USA = United States of America

**Fig 2:**
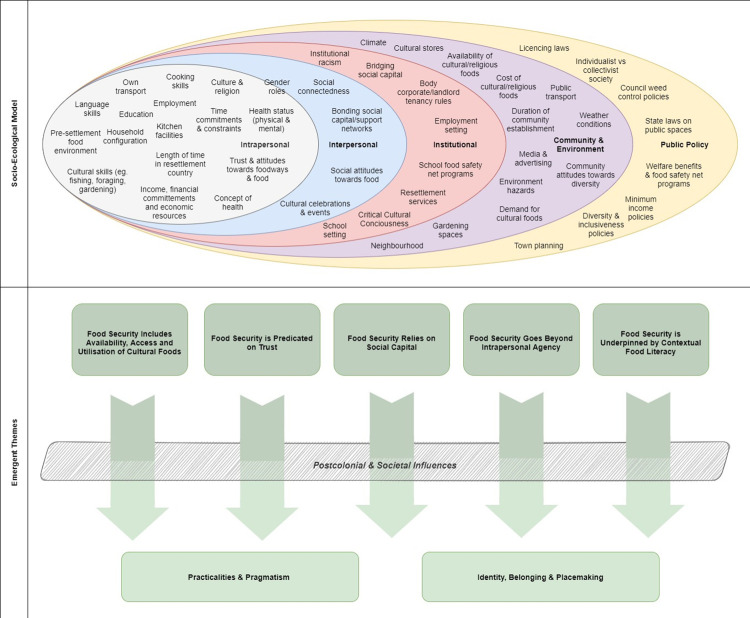
Graphical representation of findings from selected articles.

The determinants of FS for people with lived refugee experiences were found across all levels of the SEM. Two overarching themes emerged: “Identity, Belonging and Placemaking”; and “Practicalities and Pragmatism”. Five subthemes were identified which intersected across the two major themes. Analyses revealed subthemes were also influenced by “Postcolonial and Societal influences”, which was recognised as a final theme. Fig 2 provides a graphical representation of the categorisation of determinants across the SEM and the themes which emerged from the selected articles. Each theme will now be described in more detail, with quotes from the selected articles inserted at relevant area to support the findings.

### Practicalities and pragmatism

The research highlighted that people from refugee backgrounds had many barriers to FS, and consequently created practical solutions and made pragmatic decisions to improve their access to food.

To find cultural foods, people adapted to their new environment using social media and mobile phone apps to locate those foods [44, 45]. People travelled long distances, went to multiple stores, markets, community gardens, grew food in backyards, fished and foraged to obtain the variety of food they desired [5, 16, 41, 43–45, 47, 48]. Lack of and under-employment as well as low-income predictors were associated with increased HFI for people with lived refugee experiences [13, 35, 38, 49]. and therefore many strategies to improve HFS were centred around maximising limited economic resources.

*“We limit on the purchase to cheap food, so we can still have money until the end of the month. If we do not do that we will stay without money for several days until the government gives us money. We buy, for example, rice, lentils because they are cheap and they are satisfying meals” [45]*

Some people chose to retain a diet of cultural foods with a higher nutritional value which were cheaper than fresh foods, such as rice, lentils and frozen or canned vegetables [45]. People also employed strategies such as buying food in bulk, substituting foods for cheaper alternatives, bulking-out meals with cheaper ingredients to feed more people, buying foods with higher satiety value, substituting cultural foods with cheaper local foods, asking for food on credit at cultural stores or growing cultural foods [1, 13, 18, 37, 40, 41, 45, 48]. People also managed the billing cycle by purchasing cultural foods early and cheaper local alternatives later in the cycle [41, 48].

The following subthemes explore some of the factors which affected FS and led people from refugee backgrounds to develop practical solutions and pragmatic decisions to provide food for their families.

#### FS includes availability, access and utilisation of cultural foods

The significance of incorporating cultural foods into the diet of people with lived refugee experiences can be seen in the attitudes and behaviours towards non-cultural stores and foods. People were reluctant to include unfamiliar and non-cultural local foods in their diet [5, 35, 40].

*“we don’t know how to eat lamb…we eat goat, because lamb we no like” [43]*

Large supermarkets can be more accessible and cheaper; however, people often did not choose to shop there [5]. These stores did not supply cultural foods, were unfamiliar and daunting, and many with strong accents struggled to be understood [43]. Some people had trouble identifying foods in unfamiliar packaging or locating foods with different storage techniques (for example frozen compared to fresh) and were overwhelmed by the huge variety of foods available [5, 38, 43, 44]. People reported mistaking similar looking foods, such as lamb, beef, and pork, or discarding unfamiliar foods, which resulted in wasted food and money [5, 6, 43]. Instead, people often acknowledged cultural foods were expensive, but nevertheless chose to consume them [1, 34, 42]. Employment, a predictor of increased household income, was found to be associated with larger quantities of cultural vegetables available in the household [5], illustrating that people from refugee backgrounds choose to purchase cultural foods where higher income permits. Additionally, cultural stores, farmer’s markets or buying foods directly from farmers may be preferable which, although more expensive, may provide access to a larger or fresher supply of cultural and religious foods, or where shop assistants are more likely to speak their language. [1, 5, 38, 41, 42, 44, 46–48]

The selected studies often reported cultural and religious foods were expensive and difficult to locate [18, 37, 40–42, 45, 47].

*“So it [vegetables] is hard to get and very expensive. We can buy only in Asian market, Asian grocery store, so all the foods, the Asian food comes from Los Angeles you know, California and some from New York City, so they cost … [a lot]. I mean … the vegetables are very expensive.” (Karen respondent) [18]*

Given the low incomes prevalent in this population [34, 41], people with lived refugee experiences dedicated substantial time, effort and budgeting skills during food procurement, which may predispose them to FI [1, 38]. People reported travelling to multiple stores over long distances to find the variety of affordable cultural foods they desired [5, 16, 41, 43, 45, 47]. Alternatively, people also found better buying power on arrival in a high-income country, or certain cultural foods were comparatively more affordable. [42, 48]. Additionally, the high cost of fresh vegetables meant that certain foods such as meat were perceived as better value for money [42]. In these instances, people were able to incorporate a greater quantity of those cultural foods into their diet.

#### FS is predicated on trust

The perceived safety and quality of foods in high-income countries was a recurring subtheme. People were concerned foods in supermarkets were less fresh, affected by long-term food storage practices, and in cultural stores, were sold without or past their expiry date. Participants were hesitant of ingredients in packaged goods, and importation of cultural foods meant they travelled long distances, possibly impacting on perceived quality [40–42, 46, 48]. People were also concerned by soil quality, chemical residues from farming techniques, hormones used in animal products, and changes to the composition of foods during processing, impacting food safety [1, 40, 41, 46, 47].

*“I like Cambodian food because there is less fat and they use less chemicals. Here in the U.S, the chicken most of the time is injected with hormones; so they don’t grow naturally. I like fish, because it’s from the ocean and they don’t add chemicals to it.” [41]*

These factors contributed to a mistrust of the quality and safety of foods sold in high-income countries, was quoted as the reason food was perceived as tasteless [40, 42, 44], and likely affected decisions around food procurement.

Mistrust of legal systems embedded in the host country may leave people with limited options to seek help or uphold their legal rights. People reported concerns over being evicted from of rental properties, not having the same rights as other citizens, feeling they were unable to seek police assistance, and a fear of retaliation if crimes were reported [18].

*“Also the worry of [being] kick[ed] out by the landlord make us silent. Feeling that unequal right because we are not citizen[s] when we compete with the other household who are citizen[s]. Landlord doesn’t fix the house problems. Most [in the] Myanmar community like to rent the house with Myanmar landlord[s] in our community. If we see someone using … drug[s], we can’t call the cops because we can’t speak up. We don’t know where we can get … help from.” [18]*

This may lead to underreporting housing maintenance issues and faulty kitchen facilities, or apprehension when travelling to grocery stores [18, 47]. Additionally, where landlords mistrusted people with lived refugee experiences, especially regarding the ability to pay rent, it may be more difficult to find housing located near cultural food environments [18, 44]. In Canada, private sponsorship organisations sometimes did not continue providing settlement support services after a relationship breakdown with those they sponsored, impacting on housing, income and FS [45].

#### FS relies on social capital

At the interpersonal level, social support was associated with improved HFI for people from refugee backgrounds [34, 37, 39]. Community members helped improve access to food by sharing knowledge on where to find cultural foods, borrowing and lending money between themselves when resources were scarce, consolidating their financial resources to take advantage of bulk shopping opportunities or car-pooling to grocery stores [16, 34, 37, 44, 45, 47, 48].

*“I ask [my friends], I am preparing this meal, where can I find good quality?[…] Where is the quality that I can trust? Did you try it? We have a group, us Syrians in Canada on “whats” [referring to Whatsapp] and “face” [referring to Facebook] so anybody who has a question, messages in the group and others answer, like almost 50 women in Canada” [45]*

Utilisation was increased by community members teaching new arrivals about adapted cooking methods, equipment or sharing household goods [16]. Availability was improved through shared horticultural knowledge and exchanging seeds of cultural foods [43]. which also created new stable and sustainable foodways. These community actions also facilitated improved food agency for all community members. However, it is important to note that social capital was employed differently, depending on culturally acceptable behaviour. For example, there was a cultural stigma and shame attached to admitting to FI or sharing food in some cultures [36, 41] and it was more acceptable to dine with friends in others [1, 48], rather than sharing of household goods during a food crisis. Additionally, social capital was unable to protect against HFI where high levels of poverty existed and HFI was experienced by most members of the community [34].

#### FS goes beyond intrapersonal agency

There were many factors which affected FS at and above the intrapersonal level of the SEM. During the initial resettlement phase, it was found that people had competing priorities, such as learning the local language, attending orientation programs, caring for children, and finding housing and employment [40, 46]. Some people strived for financial independence however, household expenses were significantly increased at this time [13, 36, 40, 44]. Rent and utility expenses were novel for some, and there were significant expenses to establish a new household and in some cases, high medical costs [36, 42, 45, 48].

At the interpersonal level, some had an important social responsibility to provide for extended family who may have resided in another country [13, 40, 48].

*"I have to help when my mother calls, because my mother she has eight children, and they are little children in Africa, and I was the firstborn for my mom. So they call and say, “A dog bit your little brother and they take the flesh off,” and the money I was having, I send it on the same spot. So no food…” [13]*

Additionally, institutional racism may have impacted on access to employment [34, 37, 44, 47]. The combination of high household expenses and low incomes meant many people had to make pragmatic allocation decisions, and food was often designated a low priority [13, 45, 46, 48].

The initial resettlement period was identified as a time of high stress, culture shock, and mental health disturbances [6]. Some found it difficult to adjust to the new and often busy schedules where support networks, extended family and friends were sometimes absent [40, 42, 45, 46].

*“[…] they have all this stress coming on them that […] from the society’s expectations of them to learn English, to access […] to get jobs and um, have their children in school and take good care of them. There’s a lot of, like, pressure on the families that’s kind of goes on top of everything that has to do with the food.” [46]*

The level of formal education received prior to arrival by people with lived refugee experiences was found to be associated with improved HFS [1, 13, 37, 38]. Education may be informal in many countries, in areas which are not valued in the new country, such as agrarian skills, or not recognised without adequate paperwork, which limited job opportunities, predisposed people to low incomes and HFI, and impacted on their self-worth [42, 45]. When employment was obtained, it was often in minimum wage positions, which required long works hours to earn sufficient money to cover household expenses [46]. Depleted economic resources and the poverty experienced by this population was reported to lead to anxiety and worry as people waited for food assistance or pay checks while food stocks dwindled [1, 45, 48, 49]. Additionally, some people had family living in unfavourable conditions in other countries, which led to feelings of guilt, loss of appetite and the need to send already limited money overseas [36].

This client has eight children and a wife back in ‘identified nation’–he feels too ‘guilty’ to eat food [36].

These factors illustrate the bi-directional relationship between mental health and FI, where FI may lead to anxiety over obtaining enough food with insufficient financial resources, and guilt may lead to FI when people send funds overseas. Consequently, one study found a strong association between HFI and depression for people from refugee backgrounds [49].

On arrival, people with lived refugee experiences are connected to resettlement services and agencies designed to facilitate settlement. Typically, these services provide an orientation, which is seen as an important part of the resettlement process providing valuable information and potentially facilitating FS [16]. Support services decline significantly within the first two years of arrival as it is expected people will become self-sufficient [34]. Rates of child hunger, an extreme form of HFI, improved the longer people from refugee backgrounds lived in the host country [35]. However, although there are mixed results, most studies reported that less severe forms of HFI continued to remain high long after resettlement [13, 37, 38], indicating ongoing support services may be warranted. It was also reported some people were unfamiliar with support agencies, agencies were unhelpful or unwilling to listen to client concerns, and one failed to make alternative support arrangements during staff leave periods [36, 45].

*The settlement worker went on holiday and left this couple without skills to shop and manage money. They bought all food from a local deli and ran out of money. Their stove broke down and they had no way of cooking and did not know who to ask to get it fixed [36].*

This may indicate that agencies are under-funded, do not understand the needs of their clients or are unable to provide culturally appropriate services.

Having access to personal transport was associated with increased HFS for people with lived refugee experiences and, when unavailable was cited as a major barrier to FS [1, 5, 16, 18, 36, 41, 44, 47, 48]. Without a car or other means of transport people reported difficulties managing grocery bags and children, carrying groceries over long distances, and foods had to be purchased in smaller quantities [5, 16, 18]. Additionally, people were limited in where and how many stores they visited, and important cultural stores were sometimes inaccessible [1, 5, 18]. This can make it more difficult to obtain affordable and diverse cultural foods [5]. Consequently, purchasing a vehicle was sometimes prioritised by people from refugee backgrounds, and they experienced relief when it was achieved [16, 47]. However, low incomes and the financial pressures of driving lessons and car expenses may mean owning a car is unattainable for many [16, 45].

#### FS is underpinned by contextual food literacy

Orientation and resettlement services may not necessarily understand the cultural significance of foods and may instead focus on informing their clients on where to find cheap and affordable foods, which may not be culturally appropriate [6, 18, 46]. In some instances, orientation may include training on using cooking equipment and a shopping trip to a supermarket which sells cheap, and often unfamiliar, foods [6, 43]. Previously, people may have shopped at fresh markets, and consequently, shopping in unfamiliar settings like a large supermarket was described as unsettling and distressing [5, 6, 43, 46, 49]. Additionally, people with lived refugee experiences sometimes forgot information provided in orientation sessions among the large volume of other settlement information provided or because of its lack of cultural relevance [16, 47].

*“We [her family] had issues with food stamps [Supplemental Nutrition Assistance Program (SNAP)] because we couldn’t remember how to swipe or use the pin number. Sometimes we would go to the grocery store and couldn’t remember our pin number and we would have to call our caseworker to remind us what the pin number was.” [16]*

Food procurement can be difficult when the community and environment is not inclusive to the needs of residents. Difficulties navigating the food environment were associated with increased HFI for people with lived refugee experiences [1, 38]. Public transport systems and supermarkets typically provided signage in the local language which posed difficulties for many who were still learning this language [5, 45, 47]. Good language skills may also be required to take advantage of store sales or to undertake comparative shopping [45]. Food labels require literacy skills to understand the products contained in the package, the nutritional content and preparation instructions [38, 45–47]. Consequently, poor language skills were associated with HFI [13, 35, 38, 39] and were often cited as a major barrier to FS [5, 16, 45–48], and therefore learning the local language was a high priority for many [42].

*“Sometimes since I don’t know the language my heart gets tired. My mind is not happy either since I don’t know the language. Sometimes, it like my mind is just so done with it.” [18]*

Having a supermarket in the neighbourhood was associated with increased availability of cultural vegetables [5]. However, affordable housing was sometimes located in remote or low socioeconomic neighbourhoods, far away from services or grocery stores which may have increased the complexity of getting to food environments, requiring residents to catch multiple modes of public transport which may be confusing [5, 18, 36, 45, 46, 48]. Insufficient public transport connections led to some people waiting long periods at transport intersections or walking long distances to procure food, sometimes during extreme temperatures or through unsafe neighbourhoods [16, 18, 45, 46].

### Identity, belonging and placemaking

The role of food in building and maintaining identity, belonging to culture and community, and placemaking emerged as a theme.

Foods was embodied in culture and cultural identity, and provided a connection to home, people, places and between the past and present [1, 6, 43, 46].

*“When we are eating our foods we feel like we are in our country again” [1]*

This was especially important when people with lived refugee experiences arrived in high-income countries where aspects of their culture, such as language, dress and food, were not necessarily understood in the broader community [46]. Consequently, there was a strong preference to continue eating cultural foods [1, 5, 6, 34, 40, 43, 46], and people experienced a deep longing for these foods if they could not be consumed [40, 43, 46, 48].

*“We have done this for a long time. We do not want to change our culture. We want to keep our culture, eating the same stuff we were eating before” [5]*

Mealtimes were identified as an important opportunity to share culture and connect with family and friends, and people often preferred in-home and family orientated meals [1, 40]. In some cultures, food was procured daily, cultural meals were prepared over long periods, and extended family were involved in food preparation [40, 43].

The intrinsic nature of food and culture extended to procuring, preparing, and consuming food. Where food could be acquired using cultural ways, settlement was positively impacted [46]. Gardening for some cultural groups brought a connection to culture and traditions, a sense of identity, memories of home, and improved mental health [6, 43, 47].

*“When we live back there [Burma] we cultivate, and when we come here, if we don’t cultivate, it is uncomfortable for us. Cultivating is like living in our house, our country. It seems like that and it enjoyable for us.” [47]*

In contrast, some participants described their disappointment and distress at being unable to obtain foods in culturally appropriate ways [6, 40, 45, 46]. The processes of acquiring, preparing, and eating cultural foods were therefore a key element of identity, belonging and placemaking.

The following subthemes explore some of the factors which impacted on the meaning of cultural foods and affected the identity, belonging and placemaking of people with lived refugee experiences who arrived in high-income countries.

#### FS is predicated on trust

When cultural foods were not easily available or accessible, people from refugee backgrounds experienced anxiety over obtaining sufficient cultural foods [43, 45, 49]. On arrival, the change to the food environment caused a food culture shock for some [6, 43, 46], which was described by some participants as “horrific” [43].

*One participant stated that food shopping was “very scary and that it made her worry”, so only her husband went shopping for the first year [43]*

These feelings of distress and anxiety may be exacerbated when religion prescribes fundamental rules around food, and substitutions are not permissible. For example, people following Islam consume only halal meat products and other foods are strictly prohibited (haram), however, in high-income countries these may be difficult to find and expensive [35, 40, 44, 45].

*“When I buy canned food I have to read the ingredients and this is a challenge. I don’t buy anything that I don’t know. If I don’t know the ingredients or if it has gelatin or preservatives, I don’t buy it. I have to know it and this is a big challenge” [45]*

This may lead to anxiety over finding religiously sanctioned foods, and mistrust of foods and their labels sold in settings where halal identified products were not commonplace, such as supermarkets and restaurants.[35, 40, 44] Consequently, it was reported that substantial time and effort was required to ensure religious requirements were adhered to and FS was significantly affected if foods that met these requirements were not available, accessible, or identifiable [44, 45].

*“Even in the vegetables, they say ham flavour, so we have to check everything.” [47]*

#### FS relies on social capital

On arrival, people were often physically distanced from friends and family, and felt isolated with less opportunities to socialise [18, 42, 45, 47].

*“Like the most important thing is social life. I noticed that it is not available here. Like you are now in Syria, you are in a neighborhood, like: “good morning, my neighbor! Come for coffee. Come for tea.” You find yourself happy every day. Here, nothing. No social life. No interaction between people here. It is difficult how they’re living here.” [45]*

Cultural foods helped to re-establish community, family, social networks, and identity by bringing people together to share a meal [1, 6, 40, 43]. Meals for important ceremonies were often prepared as a community, where women came together to cook [6]. Food was used to pass on customs, traditions and culture through generations and strengthen family ties [18, 40]. Cross-cultural sharing of food helped create friendships outside of the cultural community and provided an opportunity for both parties to learn about each other’s culture [6, 43].

*“Yes, I am happy, so I want other people to eat our food too … other people are interested in Burmese food, I encourage them to eat our food.” [6]*

Showcasing foods at significant celebrations and ceremonies with the wider community created a greater connection to the new country and allow people to feel pride in their culture and cultural foods [6, 43]. Sharing of cultural food therefore is critical for social connection and belonging as people settle into the new country.

The intrinsic connection between food and placemaking positively altered foodways and improved FS [6]. As communities expanded and evolved over time, so did the demand for cultural foods, leading to increased supply [6]. and social capital had a key role in this process. Established community gardens became aware of the demand for particular plants through people in the community or by contacts with local shop owners [6, 43]. Seeds of cultural foods and horticultural knowledge were exchanged among community members who wished to plant foods they struggled to find elsewhere [43]. As gardens expanded, oversupply was sometimes sold at local markets, providing a source of income [43]. As communities became established, cultural foods businesses were commenced or started to stock more cultural foods [46]. These factors contributed to an increased supply of cultural foods until they became widely available [6, 46, 49]. Restaurants, which provide a source of income and an opportunity to share food and culture, were also established and cultural foods became more mainstream for the wider community [6].

*Furthermore, private gardens in homes are rapidly expanding and are providing for their community, as well as supplementing incomes by sending specialised produce, such as rosella leaves, off to market locally and interstate. Sought after seeds are exchanged amongst the community and horticultural knowledge is highly valued and passed on [43].*

A mix of reshaping the food environment and dietary acculturation influenced those who had resettled but also other residents as they began consuming new foods [6]. Placemaking and social capital reshaped the food environment, illustrating how migrant communities can influence and have sovereignty over their foodways.

#### FS is underpinned by contextual food literacy

At the community and environment level, people may be food literate in their home country, but not necessarily in the arrival country because the range of foods available, and their nutritional value and meaning can be different depending on the context. Some people who regularly experienced food shortages, strived to consume enough food for their energy needs [36, 42, 48]. On arrival in a high-income country, where there is a surfeit of food available, some people found it difficult to reframe their thinking that energy-dense foods were not necessary or unhealthy [42].

*In further discussion, participants mentioned that people in their villages often experienced food shortages and worked hard to afford 2 meals. Hence, it was difficult for them to comprehend that certain food elements could also lead to poor health [42].*

Additionally, some cultural foods were sought after for nutritional properties that were rare in the home country. For example, Henderson et al. [46] reported that ground beef was expensive and prized in Afghanistan and was vital for its iron and protein content. However, the high availability and affordability in Canada may lead to over-consumption [46]. Therefore, although it was considered healthy in Afghanistan, it could lead to an increase in saturated fat intake and negative health outcomes in Canada.

### Postcolonial and societal influences

This review identified many of the challenges to FS for people with lived refugee experiences were caused by societal expectations and norms, and political structures which were infiltrated by colonial ideas.

Changes to social networks, living environments, and school and work commitments impacted on the ability of people with lived refugee experiences to consume cultural meals in a cultural way [6, 40, 43, 46].

*In Somalia this is typically taken at lunchtime and involves the whole family. Women reported ongoing difficulties in shaping an alternative arrangement that met the dual objectives of bringing the family together and ensuring an appropriate food intake [40].*

Societies which valued self-sufficiency meant extended family members, who previously cooked together to prepare family meals, were sometimes working in the new country and unable to help prepare these meals [45]. Food preparation often changed from being a social activity with extended family to the burden of one family member, which impacted on a family’s ability to prepare and consume cultural foods [40, 45].

*One participant had a preference for a very time consuming ‘hot soup’ made from beef or goat bones. In Myanmar such a soup would be purchased from a neighbourhood stall, or would be cooked up for a whole family by a mother, grandmother or aunty. In Coffs Harbour this participant now has Weet-bix for breakfast [43].*

Societal expectations and norms in the working environment and school setting also influenced food choices away from cultural foods. In some cultures, it was typical for family members to return home at noon for a family meal [40]. In the new environment, it was expected that people purchase lunch or pack that meal and take it to school or work. However, people with lived refugee experiences often reported they were unable to eat cultural foods in these environments as they required reheating, were problematic to pack and transport in a lunchbox, or they felt uncomfortable as the foods had strong odours or looked unusual to others [40, 46].

*[Newcomers will say] “show me how to make a lasagna, a vegetarian lasagna, show me how to do, you know, more Canadian stuff.” You know, because if they take it to work, you know it doesn’t smell as much, right? [46]*

Children were particularly influenced by the pressure to fit into the social environment, and consequently, pressured parents to provide non-cultural foods which were similar to their peers [5, 40, 42, 46, 47].

Gender roles were often viewed differently in the new country, which impacted on cultural belonging, mental health and FS. In some cultures, it is typical for women to be responsible for childcare, household management or food preparation, shared among extended family members [40, 45]. However, on arrival women sometimes entered the workforce or were paid welfare entitlements directly, which switched their household position to income provider or manager [45, 46]. Without extended family support, men sometimes found themselves carrying out activities they viewed as roles for women, which negatively impacted on their self-worth and mental health [45, 46]. Additionally, they may not have adequate food preparation skills, adversely impacting on HFS [45].

*“I found that trauma is more evident in the men because they come from a culture where the man is the breadwinner and the decision-maker and the new situation emasculates them. The women come here they already didn’t know anything so they are more eager to learn and more receptive to learning and so the man has to act as capable and wants to be seen in her eyes as relevant. The child tax benefit plays a major role in conflict because it comes in the name of the woman. So for the first time this woman, who never had anything and it comes in her name. So the man feels emasculated.” [45]*

At the political level, neoliberal influences were seen in the welfare system as it provided insufficient entitlements, late payments, and eligibility criteria set too high for people with cultural needs [41, 44–46], which can worsen FS. The rules, regulations, and paperwork were found to differ depending on where you lived, change regularly, and designed for people that were proficient in the local language.

*“they [nutrition assistance program representative] sent me paperwork for reviewing, and I didn’t know what to do with this paperwork. After that I found no more money in the account.” [16]*

This also applied to other forms of income, such as establishing a business which required registration paperwork to be filed [6].

*“It’s really hard to open a shop because it requires certificate II or III, not like in India [her transition country].” [6]*

These requirements were not inclusive of people with lived refugee experiences that may have minimal formal education, poor literacy levels, lacked important documents or were still learning the local language, and therefore often struggled to complete the required forms [6, 16, 41, 42, 47, 48]. This resulted in delayed payments, cancelled entitlements, or prevented businesses opening when paperwork was not completed or processed in a timely fashion [6, 36, 41, 46, 48].

In the United States there are two important food safety nets provided by the government, Supplementary Nutrition Assistance Program (SNAP) and special supplementary program for women, infants and children (WIC). These programs are designed to supplement other government welfare entitlements to provide additional access to food and are considered reliable food safety nets [16, 41, 47]. The supplementary nature of these entitlements mean they are insufficient for all food costs, especially for a culturally appropriate diet [41]. However, other welfare entitlements were not enough to cover household expenses, and therefore many people from refugee backgrounds stretched SNAP and WIC to provide for their full cultural food budget [16]. Entitlements were often expended before the end of the pay-cycle [1, 48], and so was associated with increased HFI [13] as it left families with limited options for food purchases. If entitlements are subsequently lost due to difficult paperwork, people may struggle to afford food.

Institutions may be structured in ways that are not inclusive to the needs of people with lived refugee experiences. Orientation services include courses, such as English as a Second Language, which can improve employability and therefore income and FS status. However, these courses were found to be inflexible to competing priorities, such as childcare, and when employment was gained before the course was completed, some people found they had to choose between completing the course or losing their job [18, 37]. This choice could be seen as a decision between short-term reliance on welfare or long-term poor language skills and minimum wage positions, both of which adversely affect FS. Gaining employment was sometimes difficult as institutions often required language skills in addition to other standard skills required to undertake job responsibilities, resulting in limited job opportunities, unemployment, and low income [34, 35, 37, 44, 46].

Housing and neighbourhoods can affect FS and was also influenced by postcolonial political and social ideologies. At the public policy level, town planning was not inclusive for people with lived refugee experiences, who were overlooked in community consultations, resulting in cultural stores being located at a distance from where people live and poor public transport connections to cultural food environments [18, 47].

*“I am not able to go to the Sunday market (farmers’ market) as it is in (name of neighborhood) and I do not have a car. It is very difficult to travel on the train with food and children. Even when I go with the train, the market is far from the train station.” [5]*

Landlords often displayed stereotyping, discrimination, and racism towards people from refugee backgrounds. Some people reported homeowners were unwilling to carry out basic maintenance, leaving residents living in unacceptable and unsanitary conditions, affecting their food safety [18, 47]. Others reported landlords claimed unsubstantiated concerns about their ability to afford the rent and misunderstanding were common [18, 47].

*“In Australia we have difficulty finding a place to rent for several reasons. First, we are a big family and second, our family relies on Centrelink payment and landlords are not trusting us that we can afford their rent.” [44]*

Some found alternative housing were difficult to locate or deposits were forfeited if they left, suggesting limited choices regarding housing options [18, 47]. Some people raised concerns over being evicted, competing with other residents who have rights of citizenship, and although they may have lived in high crime neighbourhoods, they felt they were unable seek help from police or other sources [18].

Using a postcolonial lens, dietary acculturation, that is, encouraging people to adopt the diet of the host country, was a reason behind the lack of success of interventions aimed to address FI. Programs which focused on improved access to local foods, such as foodbanks and community gardens, or knowledge and skills development about local cuisine using nutritional education programs were often culturally inappropriate and did not meet the needs of people with lived refugee experiences [36, 41, 43, 45, 46]. At worst, people from refugee backgrounds experienced feelings of shame, stigma, social exclusion, and racism during these programs [36, 46].

*“The school will pick and choose kids that have difficulty in having enough food to eat. So, they send the food home with the kid, and I get the food. It’s basically the surplus of the food that school has” [41]*

Where interventions met the needs of people with lived refugee experiences and considered the significance of providing cultural foods, their value may not have been recognised and they struggled to obtain ongoing funding and were subsequently shut down [18, 46]. People with lived refugee experiences may prefer to rely on social capital for support as a more culturally appropriate way of managing HFI compared to other interventions such as foodbanks [36, 40, 48].

Cultural food gathering practices such as foraging, fishing and growing foods provide people from refugee backgrounds agency over their foodways [18, 47]. However, postcolonial structures in the form of rules, regulations, and allocation of public spaces may have inhibited the ability to obtain food in culturally appropriate ways. For example, some people were prevented from gardening due to insufficient allocation of public gardening areas, restricted gardening spaces in tenanted properties, and complex rules imposed by landlords and community garden organisations [16, 18, 43, 46, 47]; the need for licences may have prevented people from fishing due to cost and difficulties completing the paperwork [43]; state laws regarding plant removal from public spaces may have prevented foraging activities [18]; and council weed control policies or inadequate pollution control may have placed people at risk if they consumed contaminated plants [43, 47].

*One respondent noted, for example, that she would forage for plants in the urbanscape but was threatened by a police officer with a ticket for removing the plant [18].*

The rules, regulations and policies described were implemented without consideration of the needs of people with lived refugee experiences or local Indigenous populations that may have similar practices, and consequently reduced access to food and worsened FS.

## Discussion

This review has highlighted that individuals and communities displayed remarkable strength and resourcefulness when accessing food, and cultural skills were protective against FI. Individuals drew on food skills and knowledge, dedicated extraordinary effort to navigate the food environment, and supplemented their food supply by using culturally appropriate and sometimes cost-free methods [5, 16, 41, 43–45, 47, 48]. Communities supported each other by sharing their knowledge, combining resources, and teaching each other about the food environment [16, 34, 37, 44, 45, 47, 48]. Communities also positively reshaped the food environment to create new foodways and introduce cultural foods to other residents [6, 46, 49]. This review also found numerous examples of institutions, communities and environments, and public policies structures that restricted people with lived refugee experiences from enacting their agency, utilising their skills, or reaching their full potential. These structures limited people’s access to resources and adversely affected FS through political bureaucracy, and societal norms and attitudes. Some examples included racism, co-workers denunciating cultural meals, landlords failing to undertake basic maintenance, inadequate support by welfare systems, and town planning overlooking the needs of people from refugee backgrounds [18, 36, 40, 46, 47]. It is clear there is institutional racism embedded in these upstream structures, which must be addressed.

The resilience of people and communities with lived refugee experiences was identified by using a strengths-based approach. Two studies by the same author investigated the social and cultural role of food in placemaking, which is a strengths-based approach [6, 43] and one study used aspects of this approach when discussing the linguistic and cultural competency of organisations and governmental agencies to address poor language skills [39]. However, the remaining studies included in the review tended towards a deficit-based model by focusing on individual attributes, such as poor language skills, low income or an over-reliance cultural foods [1, 5, 13, 16, 18, 34–38, 40–42, 44–49]. Interventions aimed at improving FS for people with lived refugee experiences were generally designed to improve nutrition knowledge, cooking skills, or availability of food for individuals [41, 50–56], or more specifically, to correct perceived deficits of an individual’s attributes.

Some selected articles explored the healthiness of cultural foods [5, 34, 41, 42, 44, 46, 48], however, the concept of “healthy” was not explored in any of these studies and appeared to be centred around physical health. Health may be conceptualised differently depending on cultural background or the lived refugee experience. For example, one study reported that people were unsure of the healthiness of cultural foods, yet later discussed the nutritional value of particular cultural foods [46], illustrating how “healthy” is a complex construct. Cultural foods are often identified as being vital for spiritual health, building strength, giving energy and satisfaction, improving immunity against diseases and can aid in the recovery from illnesses [5, 40, 41, 43, 45, 46], and therefore a healthy choice.

A deficit-based approach is not uncommon in research, with academia historically overlooking the impact of discrimination and other upstream factors on health [15]. Previous reviews on this topic have often approached FI from this deficit-base. Lawlis and Islam [17] used the four dimensions of FS and identified the major challenges to FS for people from refugee backgrounds in Australia included low income, limited social support, maintaining traditions and understanding the “Australian way of life”. Mansour, Liamputtong and Arora [20] investigated FS among Middle Eastern and North African migrants in high-income countries and found that maintaining culture and a cultural diet increased the likelihood of FI. These findings suggest assimilation may be necessary to enable access to resources and improve FS. Alternatively, it could be argued that FI was adversely affected by the neoliberal and colonial ideologies through social and political structures and it is these structures that must be the focus of future policy changes and interventions to improve FS for people with lived refugee experiences.

This review was underpinned by a rigorous search strategy and methodology approach. It does, however, have some limitations. The selected articles were all conducted in three countries (United States, Australia, and Canada) and therefore the findings may not be generalisable to other high-income countries. All articles were included in the review regardless of their quality assessment. However, the contribution from those of lower quality were supported by higher quality articles, and therefore were relevant.

## Conclusions

The findings from this review highlight the complex and interrelated factors at all levels of the socio-ecological model that affect FS for people with lived refugee experiences. Governments, organisations and institutions need to acknowledge the fundamental influence of prevailing political and social structures and the limitations they place on the agency of people and communities from refugee backgrounds to integrate their cultural foods into their diets. Cultural foods are instrumental to the identity, culture, and spiritual and physical health of people with lived refugee experiences. Policy makers should strive for policy changes that actively work to embrace cultivate inclusivity and remove bureaucracy across all levels of government and government action. Additionally, public health workers, clinicians, community support workers, case workers, and all organisations and institutions should endeavour to work in collaboration with cultural communities, appreciate their strengths, and recognise the role of social and cultural capital and the importance of accessing cultural foods.

## Supporting information

S1 Checklist PRISMA checklist. (DOCX)

S1 Table Example health database search results. (DOCX)

S2 Table Quality assessment results of selected articles (n = 22) from systematic review. (DOCX)

S3 Table Details of selected articles (n = 22) included in the systematic review and synthesis. Avg = Average, Yrs = Years, Mths = Months.
(DOCX)
