# Supplementary material for: Determinants of food security among people from refugee backgrounds resettled in high-income countries: A systematic review and thematic synthesis
Source: PLoS One. 2022 Jun 2;17(6):e0268830. doi: 10.1371/journal.pone.0268830 (PMC9162305; doi:10.1371/journal.pone.0268830)
Supplement: S1 Table — (DOCX) [file pone.0268830.s002.docx]

# S1 Table – Example health database search results

|  | **Pubmed search strategy** | **Web of Science search strategy** |
| --- | --- | --- |
| Last search performed | 06/04/2021 | 06/04/2021 |
| Filter | Publication year 2020 to 2021, Language English | Publication year 2020 to 2021, Language English |
| Boolean search string | (Refugee*[Title/Abstract] OR "asylum-seeker*"[Title/Abstract] OR "stateless person*"[Title/Abstract] OR "stateless people"[Title/Abstract] OR newcomer[Title/Abstract] OR Refugees[MeSH]) AND Food[Title/Abstract] AND (secur*[Title/Abstract] OR insecur*[Title/Abstract] OR availab*[Title/Abstract] OR unavailab*[Title/Abstract] OR access*[Title/Abstract] OR inaccess*[Title/Abstract] OR stab*[Title/Abstract] OR utilisation[Title/Abstract] OR utilization[Title/Abstract] OR sufficien*[Title/Abstract] OR insufficien*[Title/Abstract] OR affordab*[Title/Abstract] OR unaffordab*[Title/Abstract] OR expenditure[Title/Abstract] OR literac*[Title/Abstract] OR poverty[Title/Abstract] OR barrier*[Title/Abstract] OR desert[Title/Abstract] OR "food supply"[MeSH]) | TS=((Refugee* OR "asylum-seeker*" OR "stateless person*" OR "stateless people" OR newcomer) AND (Food NEAR/2 (secur* OR insecur* OR availab* OR unavailab* OR access* OR inaccess* OR stab* OR utili?ation OR sufficien* OR insufficien* OR affordab* OR unaffordab* OR expenditure OR literac* OR poverty OR barrier* OR desert))) |
| Search results | 43 articles | 58 articles |
