# Supplementary material for: Determinants of food security among people from refugee backgrounds resettled in high-income countries: A systematic review and thematic synthesis
Source: PLoS One. 2022 Jun 2;17(6):e0268830. doi: 10.1371/journal.pone.0268830 (PMC9162305; doi:10.1371/journal.pone.0268830)
Supplement: S2 Table — (DOCX) [file pone.0268830.s003.docx]

# S2 Table - Quality assessment results of selected articles (n=22) from systematic review

| Author | Study Type | Qualitative assessment* | | | Quantitative Assessment** |
| --- | --- | --- | --- | --- | --- |
|  |  | Quality of Reporting | Sufficiency of the Strategies | Appropriateness of the Study Methods |  |
| Quantitative Studies (n=7) | | | | | |
| Anderson et al.^37^ | Quantitative | N/A | N/A | N/A | Fair |
| Dharod and Croom^38^ | Quantitative | N/A | N/A | N/A | Good |
| Gallegos et al.^39^ | Quantitative | N/A | N/A | N/A | Fair |
| Gichunge et al.^40^ | Quantitative | N/A | N/A | N/A | Fair |
| Hadley and Sellen^1^ | Quantitative | N/A | N/A | N/A | Good |
| Hadley et al.^41^ | Quantitative | N/A | N/A | N/A | Good |
| Vu et al.^42^ | Quantitative | N/A | N/A | N/A | Fair |
| Qualitative Studies (n=9) | | | | | |
| Burns et al.^43^ | Qualitative | Fair | Fair | Fair | N/A |
| Cordeiro et al.^44^ | Qualitative | Fair | Fair | Fair | N/A |
| Dharod, Xin, et al.^45^ | Qualitative | Fair | Fair | Fair | N/A |
| Hughes^46^ | Qualitative | Fair | Good | Good | N/A |
| Hughes^6^ | Qualitative | Fair | Good | Good | N/A |
| Judelsohn et al.^21^ | Qualitative (case study portion of paper only) | Fair | Good | Fair | N/A |
| Kavian et al.^47^ | Qualitative | Good | Good | Good | N/A |
| McElrone et al.^19^ | Qualitative | Good | Good | Fair | N/A |
| Vatanparast et al.^48^ | Qualitative | Fair | Fair | Poor | N/A |
| Mixed Methods Studies (n=6) | | | | | |
| Gichunge et al.^5^ | Mixed Methods | Fair | Poor | Poor | Fair |
| Hadley et al.^13^ | Mixed Methods | Poor | Poor | Fair | Fair |
| Henderson et al.^49^ | Mixed methods (only qualitative relates) | Good | Good | Fair | N/A |
| Judelsohn et al.^50^ | Mixed methods (only qualitative relates) | Fair | Good | Fair | N/A |
| Nunnery and Dharod^51^ | Mixed methods (only qualitative relates) | Fair | Fair | Good | N/A |
| Peterman et al.^17^ | Mixed methods | Fair | Poor | Fair | Good |

* Assessed using the modified Thomas and Harden quality assessment tool

** Assessed using the JBI critical appraisal checklist for case studies
