# Supplementary material for: Determinants of food security among people from refugee backgrounds resettled in high-income countries: A systematic review and thematic synthesis
Source: PLoS One. 2022 Jun 2;17(6):e0268830. doi: 10.1371/journal.pone.0268830 (PMC9162305; doi:10.1371/journal.pone.0268830)
Supplement: S3 Table — Avg = Average, Yrs = Years, Mths = Months. (DOCX) [file pone.0268830.s004.docx]

# S3 Table - Details of selected articles (n = 22) included in the systematic review and synthesis

| **Author** | **Study Design, location and dates** | **Stated Aim/Objectives** | **Community Consultation Methods** | **Sample Characteristics** | **Data Collection Tools & Methods** | **Data Analysis** |
| --- | --- | --- | --- | --- | --- | --- |
| **Quantitative Studies (n=7)** | | | | | | |
| Anderson et al. (2014)^37^ | **Design:** Quantitative, exploratory cross-sectional  **Location:** Atlanta, United States  **Dates**: Jul to October 2002 | 1. To estimate the prevalence and severity of HFI among a category of refugee families of specific concern to support resettlement support agencies and local health services at the time of the study 2. To examine the relationship between HFI and refugee mothers’ and other caregivers’ self-reported food intake 3. To test the hypotheses that vulnerability to HFI status is reduced among resettled refugees by utilization of social support, attitudes toward traditional foods, and greater income | Project conducted in partnership with community representatives. Questionnaire designed using participatory approach involving project coordinators, interviewers of Sudanese origin undergoing training (recruited through local refugee outreach organizations), and selected members of the target community. | **Sample size**: 49 for data analysis **Ethnicity**: Sudanese  **Immigration Status**: Not stated **Sex**: Not stated **Time since resettlement**: 29% 0-2yrs (n=14), 61% 2-4 yrs (n=30), 10.2% 4-5yrs (n=5) **Inclusion/Exclusions**: Self-identified caregiver with  1. ≥ one child under 3 2. a legally resettled Sudanese parent in the United States 3. parent legally resettled United States resident for < 5 yrs  **Recruitment**: Purposive from voluntary resettlement agencies, churches and other community group meetings and snowball sampling | **Tool:** Questionnaire developed with assistance from interviewers, pretested and shortened so it could be administered in <2hrs. Semi-qualitative food consumption questionnaire using FFQ, developed by study team, piloted for face and construct validity (not validated)  **Method**: Interview **Details**: Self-identified caregiver in each household interviewed during home visit conducted in Arabic, Nuer, or Dinka **Facilitator**: Bi-lingual interviewers from refugee outreach organisations of Sudanese origin. Trained in study protocol and to administer questionnaire. **Data collected**: Demographic indicators for each index child and caregiver; household, health and budget; diet and shopping; household food security; baby feeding and birth; and social life | Student’s t-tests; F-tests; chi-square tests.  Significance p<0.10 |
| Dharod and Croom (2010)^38^ | **Design:** Quantitative cross-sectional  **Location:** Maine, United States  **Dates:** October 2006 to December 2007 | To examine the prevalence of child hunger and to assess the relationship of child hunger to dietary intake and BMI in refugee children from Somalia. As a secondary objective, sociodemographic risk factors of child hunger are examined | Research project was a collaboration between the University of Southern Maine and St. Mary’s Regional Medical Center in Lewiston, Maine.  Survey instrument reviewed by three bilingual Somali health workers to develop the culturally appropriate survey questionnaire. | **Sample size**: 180 (data analysis) **Ethnicity**: Somali **Immigration Status**: Not stated **Sex**: Female **Time since resettlement**: 56% 1-3 yrs (n=109), 22% 4-6 yrs (n=43), 22% ≥ 7 yrs (n=43) **Inclusion/Exclusions**: 1. Somali women residing in Lewiston area 2. main meal preparers of the household 3. Mother of ≥ 1 child 2 to 12 yrs old  **Recruitment:** Not stated | **Tool:** Survey developed from other validated tools and reviewed by bilingual health workers. Translated and back translated by independent party. Pilot study conducted (n=35)  **Method**: Interview **Details**: Conducted at participants home in preferred language. Participants were paid US$15 **Facilitator**: Bilingual Somali women. Trained on research protocol, selection criteria, anthropometric measurements and confidentiality of data **Data collected**: FI, sociodemographic, dietary habits, anthropometric measurements | Chi-square test; logistic regression |
| Gallegos et al. (2008)^39^ | **Design:** Quantitative exploratory cross-sectional  **Location:** Perth, Australia  **Dates:** 2002 to 2003 (9mth period) | Investigate the level of and reasons for FI in a convenience sample of refugees accessing early intervention services for humanitarian entrants. | Survey conducted by Early Intervention Team (EIT) at Association for Services to Torture and Trauma Survivors, who provide service to people in Australia <12 mths. EIT members chosen as they had regular contact with clients and often previously established relationships. EIT members had discretion over participants, due to potential mental health issues.  Survey developed based on information collected during prior discussions with the target group and service providers | **Sample size**: 51 (of which 36 answered second question) **Ethnicity**: Afghanistan (n=8), Middle East/Iran/Iraq (n=15), West Africa (n=3), Horn of Africa (n=6), North-East Africa (n=9), Former Yugoslavia (n=10) *Qualitative distribution not stated* **Immigration Status**: 67% humanitarian entrants, 28% temporary protection visas, 6% not stated classification *Qualitative distribution not stated* **Sex**: Not stated **Time since resettlement**: <12 mths **Other Inclusion/Exclusions**: None  **Recruitment:** Convenience sampling (selected by early intervention team (EIT)) | **Quantitative: Tool:** Information relates to last question in food security questionnaire - causes of FI. Answer options were pre-set categorial list (including other), developed based on information collected during prior discussions with the target group and service providers  **Method**: Interview **Details**: EIT workers given discretion to choose when/where to conduct the questionnaire. Some for every new client others after establishing relationship with client. Instructions sheet provided to EIT workers. EIT workers asked not to prompt for this question. Not stated what language delivered **Facilitators**: EIT workers (case workers) with assistance of accredited interpreters if required. No statement regarding training **Data collected**: Socio-demographic details, presence of FI, possible reasons for running out of food and any other comments regarding the issue. | **Quantitative**:  Chi-square test Q2: Grouped into categories and frequency reported |
| Gichunge et al. (2015)^40^ | **Design:** Quantitative cross-sectional South-  **Location:** East Queensland, Australia  **Dates:** April to December 2012 | Assess the interaction between FI, social support, and vegetable intake among resettled African refugees | None stated | **Sample size**: 71 household (383 household members) **Ethnicity**: Burundi 75% (n=53), Rwanda 13% (n=9), and the Democratic Republic of Congo 13% (n=9) (Africa Great Lakes region)  **Immigration Status**: Not stated **Sex**: 89% Female (n=63), 11% male (n=8) **Time since resettlement**: Avg of 4.9yrs **Inclusion/Exclusions**: Primary food preparers with 1. a child under 18 yrs of age  2. the primary food preparer speaks English or Swahili  **Recruitment:** Purposive recruited from African churches, community meetings and settlement agencies then snowballing | **Tool:** Questionnaire details not provided  **Method**: Interview **Details**: No details provided on data collection except one of the interview languages was Swahili. Participants received AUD$25 grocery voucher. **Facilitators**: Researcher. No statement regarding training **Data collected**: Demographic characteristics, household food security measurement, food frequency questionnaire | Chi-square; t-test; Mann-Whitney test; Logistic regression |
| Hadley and Sellen (2006)^1^ | **Design:** Quantitative cross-sectional pilot study  **Location:** North-Eastern United States  **Dates:** Not stated | Primary objectives were to estimate the prevalence of FI and child hunger among a convenience sample, and assess the internal validity of a food security and hunger scale. Secondary objectives were to explore associations between the occurrence of child hunger and measures of household economic success and acculturation. | Qualitative research informed data collection tool: team member sat in on weekly health meetings held at a local resettlement centre, conducted two focus groups on dietary practices, and held in-depth interviews with refugees, a nurse, and caseworkers active in the community. | **Sample size**: 33 **Ethnicity**: Liberian **Immigration Status**: Refugee or asylum status **Sex**: Female **Time since resettlement**: <5yrs **Inclusion/Exclusions**: 1. Mother of Liberian origin and refugee or asylee status  2. Living in the United States < 5 yeas 3. Currently caring for a child < 5 yrs old Effort was made by the interviewers to locate both employed and unemployed respondents  **Recruitment:** Convenience sample recruited by word of mouth at the resettlement centre and in several community groups | **Tool:** Structured questionnaire developed from observations of weekly health meetings at resettlement centre, focus groups and interviews of refugees, nurse and case workers, pre-tested in three subjects.  **Method**: Interview **Details**: Conducted privately at resettlement centre or at home. Participants provided US$10. Language not stated **Facilitators**: Liberian women well known in community. Trained in interviewing skills and research ethics **Data collected**: Socio-demographics, migration history, social support, food security, dietary intake, shopping patterns, and acculturation. | Chi-square; t-test; Spearman’s correlation; logistic regression |
| Hadley et al. (2010)^41^ | **Design:** Quantitative cross-sectional (food security status collected 6 mths later)  **Location:** Mid-Western United States  **Dates:** 2006 | To examine both income and non-income factors associated with FI in a sample of refugees resettled in the United States | An exploratory ethnographic approach to ensure instruments were locally and culturally appropriate. Data collection instruments used to carry out interviews and focus groups to ensure they were appropriate. Interviewed members of resettlement agency staff (management and caseworkers), community leaders, and newly arrived refugees.  This identified topics for survey | **Sample size**: 281 **Ethnicity**: Sierra Leone, Liberia, Ghana, Somalia, Togo, and Meskhetian Turk (% not stated) **Immigration Status**: Refugee status **Sex**: 64% female, 36% male **Time since resettlement**: Avg of 48 mths **Inclusion/Exclusions**: 1. having refugee status 2. ≥ 18 yrs old  **Recruitment**: Initially recruited through a local resettlement agency then snowballing | **Tool:** Ethnographically designed questionnaire developed based on focus group, interview of target population and resettlement staff (management & case workers), newly arrived refugees, community leaders, piloted and pre-tested in several languages  **Method**: Interview **Details**: Conducted in participants language  **Facilitator**: Interviewer of same gender and spoke same language. Trained, but details not provided. **Data collected**: Demographics, difficulty navigating the food-related environment, food security | Chi-square; Spearman’s correlation; logistic regression; generalized estimating equation (GEE) |
| Vu et al. (2020)^42^ | **Design:** Quantitative cross sectional  **Location:** Atlanta, GA, United States  **Dates:** September 2017 to April 2018 | To examine FI and its associations with various indicators of acculturation (i.e., Vancouver Index of Acculturation, English language fluency, and percentage of life in the United States) and social connectedness (i.e., religious attendance and social isolation) among a diverse sample of immigrants and refugees in metropolitan Atlanta. | Community-engaged approach - partnered with two community-based organizations serving immigrant and refugee populations. Partners provided feedback on survey instruments, advised the research team on target populations, translated the instruments to appropriate languages, and assisted with data collection and interpretation of results. | **Sample size**: 162  **Ethnicity**: 51.9% (n = 84) Vietnamese, 16.0% (n = 26) Hispanic, 15.4% (n = 25) Burmese, 14.8% (n = 24) Bhutanese or Nepali, 1.2% (n = 2) Bengali,  and 0.6% (n = 1) Cambodian.  **Immigration Status**: Not stated  **Sex**: Mixed 34% (n=55) male & 66% (n=107) female  **Time since resettlement**: Not stated  **Inclusion/Exclusions**: None stated  **Recruitment**: Convenience sampling. 54.4% (n = 98) were recruited from two community health fairs sponsored by one of the partner organizations. The remaining participants (n = 82) were recruited from clients using services at the other partner organization. | **Tool**: Survey instrument, partners provided feedback on survey instruments, no further information provided on piloting of tool  **Method**: Survey  **Details**: Surveys administered during the fair or at a personally convenient time and later returned the surveys. Surveys in English, Vietnamese, Spanish, Burmese, or Nepali. Fair participants were entered into a draw for US$50/$100 gift card, other participants provided US$5 incentive.  **Facilitators**: Verbal informed consent was obtained prior to survey administration by study team members who were fluent in English, Vietnamese, Spanish,  Burmese, or Nepali.  **Data collected**: FI, acculturation, social connectedness, substance use (e.g., tobacco and alcohol), preventive care utilization, vaccinations (e.g., hepatitis B vaccination and human papillomavirus vaccination), and other sociodemographic information. | Logistic regression |
| **Qualitative Studies (n=9)** | | | | | | |
| Burns et al. (2000)^43^ | **Design:** Qualitative consultative cross-sectional  **Methodology:** Not stated  **Location:** Melbourne, Australia  **Dates:** Not stated | 1. Establish key food patterns, preferences and beliefs;  2. Identify significant food and nutrition issues in the settlement period; and 3. Identify ways of addressing issues of concern that are culturally relevant to new arrivals. | Project steering committee included representatives from Victorian Health Promotion Foundation, School of Health Sciences at Deakin University, and community health centres at Darebin, the Western Region and Springvale (resettlement areas).  Initial meeting held with key Somali workers to establish broad issues of concern, planned consultative process was culturally acceptable, and to solicit their support | **Sample size**: 33 **Ethnicity**: Somalian **Immigration Status**: All refugees **Sex**: 3 men & 30 women **Time since resettlement**: 4mths to 3 yrs **Other Inclusion/Exclusions**: none  **Recruitment:** Snowballing through Somali workers and members of the Somali community. | **Tool:** Key questions devised by the steering group (representatives of VFST, VicHealth, Deakin University, and community health centres) in consultation with health and ethnic workers  **Method**: Focus Groups **Details**: 4 gender specific focus groups held at 3 Somalian community locations.  **Facilitators**: Somali worker, nutritionist & project worker (gender unknown). No statement regarding training **Data collected (topics)**: food beliefs and practices, issues relating to children's food habits, food supply, food preparation and changes in food related issues that had occurred since arrival in Australia. **Other**: Interviews not relevant | Thematic analysis **Transcription**: Focus groups recorded and transcribed by project worker, and draft copies were verified as accurate by the other facilitators. **Analysis**: Not stated |
| Cordeiro et al. (2018)^44^ | **Design:** Qualitative cross-sectional  **Methodology:** Community based participatory research  **Location**: Lowell, Massachusetts, United States.  **Dates:** Not stated | Food security and access to healthful, cultural food in the context of safety net participation among Cambodians and Brazilians living in the post-industrial city Address the research gap on: 1. Food access among immigrant communities and their participation in federal safety net programs intended to supplement household food budgets; and  2. The impact of national and state program policies on food access among immigrants | This community-based participatory research was supported by a partnership with the Cambodian Mutual Assistance Association (org to support resettlement of Cambodians) of Greater Lowell, Inc., and the Frederick Assad Abisi Adult Education Center (provides ESL classes). The community-academic team drafted research and focus group questions, piloted the focus group questions, recruited participants, and collected data | **Sample size**: 84 **Ethnicity**: Self-identified Cambodian (n=49 + youths) & Brazilian (n=16)  **Immigration Status**: Arrival in the United State as refugee/immigrant or child of refugee/immigrant **Sex**: Mixed **Time since resettlement**: Not stated **Other Inclusion/Exclusions**: ≥ 15 yrs old  **Recruitment:** Convenience sampling (word of mouth and through local media programs, schools, community events, extension courses and community partners that serve low-income populations) | **Tool:** Moderator guides (ie questions) developed by the community-academic team. Pilot tested with key informants (i.e. community partner staff), and modified accordingly before use.  **Method**: Focus groups **Details**: 11 focus groups, including specific groups for adolescents, elderly, parents of children with disabilities, working parents, pregnant women, and ESOL learners. All held in community settings and lasted 75–120 min **Facilitators**: Adult groups held in Khmer or Portuguese with English-speaking moderators and community translators, youth groups held in English. No statement regarding training **Data collected (topics)**: Personal and intergenerational experiences with food access and food security; safety net use; barriers to purchasing and consuming healthy food; and access to, and use of, community resources | Thematic Analysis **Transcription**: Recorded and transcribed verbatim and entered into NVivo for analysis, grouped by ethnic group **Analysis**: 1. Two broad categories (healthful cultural foods and safety net programs) 2. Identified themes and sub-themes for key concepts 3. Converging and diverging themes across the categories |
| Dharod, Xin, et al. (2013)^45^ | **Design:** Qualitative exploratory cross-sectional  **Methodology:** Not stated  **Location:** Guilford County, North Carolina, United States  **Dates:**  ***Phase I*** - June 2010 to August 2011 ***Phase II*** - December 2011 to April 2012 | To understand what lifestyle and food-related differences refugees experience upon resettlement in the United States. | Phase I included recruitment and interviews conducted by 3 female community outreach workers representing participating refugee groups Except for 2 themes related to food shopping habits, all themes were verified and validated by the outreach workers and were included in the results | ***Phase I*** | | Not stated **Transcription**: Interviews recorded and transcribed verbatim. Transcripts in English were used directly for evaluation, and those in other languages were translated into English before data analysis. **Analysis**:  1. Transcripts reviewed individually to identify main themes (1. lifestyle differences and 2. differences in food environment and changes in dietary habits).  2. Codes identified and broader themes shortlisted.  3. Grouping of codes by main topics. 4. Emerged themes shared with community outreach workers for validation |
|  |  |  |  | **Sample size**: 18 **Ethnicity**: Sudan, Liberia and Vietnam **Immigration Status**: not stated **Sex**: Women **Time since resettlement**: Avg 7 yrs **Inclusion/Exclusions**: 1. Being a refugee mother of at least one child 12 yrs old or younger.  2. Being the main meal preparer for the household.  3. Currently living in Guilford County, North Carolina.  **Recruitment:** Snowballing (through personal contacts, networking or introduction by original participants). | **Tool:** Semi structured questionnaire  **Method**: Interview **Details**: At participants home lasted 30 mins & recorded. Liberian in English and Sudanese in Arabic, Vietnamese in tribal language. Participants received US$75 gift card for local grocery store. **Facilitator**:3 female community outreach workers proficient in English and native language of participant in presence of principal investigator. No statement regarding training **Data collected**: *Quantitative*: socio demographics, what specific food item increased. *Qualitative*: lifestyle changes, and difference in food environment and dietary habits |  |
|  |  |  |  | ***Phase II*** | |  |
|  |  |  |  | **Sample size**: 5 **Ethnicity**: Montagnard  **Immigration Status**: Not stated **Sex**: Mixed **Time since resettlement**: 6 yrs **Inclusion/Exclusions**: 1. Held a medical degree  2. Previously practiced medicine in their country of origin  **Recruitment:** Recruited through community contacts developed through collaboration and building connections via community-engaged scholarship. | **Method**: Interview **Details**: Conducted at participants home or community site (eg Asian restaurant). Conducted in English. Participants received US$40 gift card **Facilitator**: Not stated **Data collected**: Participants medical training history, lifestyle and food-related challenges that community members experienced in United States. |  |
| Hughes (2015)^46^ | **Design:** Qualitative ethnographic  **Methodology:** Ethnography and participatory research  **Location:** Coffs Harbour, Australia  **Dates:** Not stated | To explore and document the foodways of humanitarian migrants by investigating the social and cultural factors that enhance, or inhibit, the consumption of culturally meaningful, nutritious and desirable food | Ethnographic study requiring the researcher to embed herself into the community. In the initial stages, consulted key informants with experience working with people from refugee backgrounds, in particular those with close connections to the Myanmar community through educational, health, religious or settlement affiliations. Participatory and collaborative approach where community consultation and liaison has become an integral part of progressing the project. Participants recruited after extensive consultation with community leaders and members of the Myanmar community association. | **Sample size**: Anticipated 12-15 adult community members **Ethnicity**: Myanmar **Immigration Status**: Not stated **Sex**: mixed **Time since resettlement**: Not stated **Inclusion/Exclusions**: None stated  **Recruitment:** Through connections made in ethnographic process | **Tool:** Details not provided  **Method:** Interviews (formal and informal) **Details:** With individuals, families and small groups **Method:** patient observations **Details**: natural settings including homes, gardens and community events **Method:** documentary **Details**: depicting several participants food journeys **Facilitator**: ethnographic researcher (no information provided). Training: conducted as part of PhD **Data collected**: no specifics provided | Not yet completed - this study represents initial data |
| Hughes (2019)^6^ | **Design:** Qualitative ethnographic  **Methodology:** Focused ethnography with participatory methods  **Location:** Coffs Harbour, Australia  Period of 10 month  **Dates:** Not specified | To document the food stories of humanitarian migrants from Myanmar to understand the social and cultural factors that influence food choices in an unfamiliar environment | Ethnographic approach.  A reciprocal interview style was achieved through developing positive relationships with participants over time and adopting a conversational, empathetic tone which reflected genuine interest in wanting to hear their stories. | **Sample size**: 12 main contributors **Ethnicity**: Myanmar **Immigration Status**: not stated **Sex**: not stated **Time since resettlement**: not stated **Inclusion/Exclusions**: none stated  **Recruitment:** Purposeful and snowball sampling | **Tool:** Details not provided  **Method:** Community consultation sessions, walking interviews/home and garden tours, semi-structured and informal interviews with participants, and participant observation, including watching, talking and doing  **Details:** Observations were recorded in a field journal and were filmed where appropriate consent was provided. Key findings were used to create a documentary film depicting the participants’ food journeys. Most interviews conducted in Burmese (common language) **Facilitator**: Ethnographic researcher (no information provided). Training: conducted as part of PhD  **Data collected**: No specifics provided | Thematic analysis **Transcription**: not stated **Analysis**: Combination of the following three approaches: 1. A holistic approach that examines the entire text to elucidate themes  2. A selective approach that requires multiple readings to identify specific statements or phrases that carry meaning about the selected phenomenon  3. A detailed approach that closely examines specific sentences to provide insight into the lived experience |
| Judelsohn et al. (2020)^21^ | **Design:** Qualitative (case study portion of paper only) cross-sectional  **Methodology:** Participatory action research  **Location:** Buffalo, NY, United States  **Dates:** Not stated | To explore how municipal planning is addressing food equity for new Americans - uses same dataset as above | Data for the case study of Buffalo come from a transdisciplinary research project, DDFAR (see above). Member checking with CAG and focus group participants of findings | **Sample size**: 28 interviews (dataset from Judelsohn et al (2017)) + 1 focus group **Ethnicity**: Burmese **Immigration Status**: Not stated (see Judelsohn et al (2017)) **Sex**: Not stated (see Judelsohn et al (2017)) **Time since resettlement**: Not stated (see Judelsohn et al (2017)) **Inclusion/Exclusions**: Not stated (see Judelsohn et al (2017))  **Recruitment:** Snowballing recruited by CAG members and research team members (ties to Burmese community) through word of mouth and fliers | **Tool:** Questions developed through a community advisory group (CAG) comprised of community members and NFP orgs.  **Method**: Interview (see Judelsohn et al (2017)) and Focus Groups (in addition to Judelsohn et al (2017)) **Details**: Conducted in preferred language by interpreter (Burmese, Karen or English) **Facilitator**: Not stated (see Judelsohn et al (2017)) **Data collected**: Resettlement experience, especially pertaining to food and health experiences | Not stated **Transcription**: Translated and transcribed into English. **Analysis**: Coding using codebook with priori codes based on existing literature and emergent themes added as required. |
| Kavian et al. (2020)^47^ | **Design:** Qualitative cross-sectional  **Methodology:** Social determinants of health (SDOH)  **Location:** Adelaide, Australia  **Dates:** May to September 2017 | To explore the migration journey and food experiences of Afghani women refugees (now resident in Adelaide) in both their transition and final destination countries | Researcher is native Dari. A number of consultations with community leaders were undertaken, through the Middle Eastern Communities Council of South Australia, in order for them to understand the benefit of the study for their members and ultimately to endorse it. Once endorsed, several locally based organizations were approached, including educational and multicultural organizations working with young refugee people, and adult English learning institutions in a locality with a relatively high refugee population.  Summaries of interviews discussed in team meetings as part of data analysis process | **Sample size**: 10 **Ethnicity**: Afghani **Immigration Status**: Refugees (some on humanitarian vias and some sponsored by spouse) **Sex**: Female **Time since resettlement**: <2yrs **Inclusion/Exclusions**: Afghani female refugees who lived in Australia for 2 yrs or less  **Recruitment:** Snowballing using various strategies with numerous orgs working with refugees. | **Tool:** Open ended questions adapted from original study designed by collaborators in Canada.  **Method**: Interview **Details**: Convenient time and place eg private space is local library in participants native language **Facilitator**: Principal investigator who is bilingual in Farsi/Dari and English. No statement regarding training **Data collected**: the social determinants of health in both the transition country and Australia  **Other Methods**: Field Notes **Data collected:** non-verbal communication and immediate sense of data emerging from each interview | Grounded Theory **Transcription**: Audio recorded. Transcribed and translated by principal investigator **Analysis**: Occurred during data collection process. Prior to coding - Listened in Dari to understand core meaning, summarised and presented to project team, development of initial coding frame, fully translated and transcribed.  Coding - open coding (breaking down, examining, comparing, conceptualising and categorising data) and focused coding (grouping). Cross coded by two team members. Themes informed content of subsequent interviews. |
| McElrone et al. (2019)^19^ | **Design:** Qualitative cross-sectional  **Methodology:** Socio-ecological model framework  **Location:** South-eastern United States  **Dates:** December 2017 to February 2018 | To identify the perceived dietary acculturation barriers and facilitators to FS among female Burundian and Congolese refugees living near a mid-sized city in the south-eastern region of the United States | None discussed | **Sample size**: 18 **Ethnicity**: 67% Burundian (n=12) and 33% Congolese (n=6) **Immigration Status**: Self-reported refugee **Sex**: Female **Time since resettlement**: Avg of 67mths (5.6yrs) **Inclusion/Exclusions**: Female, 18 yrs of age or older, self-reported refugee status, and native of a Sub-Saharan African country  **Recruitment:** Word of mouth through refugee programs using network then snowballing | **Tool:** Semi-structured interview guide. Tested on 3 members of target refugee community for wording, content and cultural relevance.  **Method**: Interviews **Details**: Conducted at participants preferred location. Interpreter translated to participants preferred language. Participants were provided US$25 gift card **Facilitator**: Principal Investigator with aid of interpreter. No statement regarding training **Data collected**: Post resettlement FS  experiences regarding culturally familiar food access, food shopping, transportation to food outlets, meal preparation habits and cooking methods/equipment, and government nutrition assistance programs. Sociodemographic information  **Other methods**: Field notes | Multi-stage, iterative data-driven analysis process to identify patterns and themes and constant comparative method (grounded theory approach) **Transcription**: Audio recorded **Analysis**: Use of Socio-ecological model framework + household added. Upload transcripts to NVivo 2, collaboratively created codebook (iteratively updated), multiple stages of analysis by research team. |
| Vatanparast et al. (2020)^48^ | **Design:** Qualitative cross sectional  **Methodology:** Not stated  **Location:** Toronto & Sakatoon, Canada  **Dates:** December 2016 to February 2017 | To identify the FS status of Syrian refugee families in Toronto and Saskatoon before and after their arrival, together with the capacity of support systems to address their food needs.  In addition, the study engaged in an in-depth account of how culture, socio-economic status and gender impact the level and management of FS for Syrian refugee families | None discussed | **Interviews with Syrian refugees**  **Sample size**: 54  **Ethnicity**: Syrian  **Immigration Status**: All refugee status, either private, govt or blended sponsorship  **Sex**: Mixed 70% male (n=38), 30% female (n=16)  **Time since resettlement**: <3 yrs  **Inclusion/Exclusions**: Resettled in two Canadian urban contexts, Toronto and Saskatoon, since November 2015.  **Recruitment:** Non-probability snowball sampling and data saturation determined sample size. Contacts and connections with local refugee settlement agencies and community members were leveraged to recruit participants  **Interviews with key informants**  **Sample size:** 15  **Inclusion/Exclusions:** Key informants responsible for the implementation of refugee programming  **Recruitment:** Range of settlement and community-based organizations (settlement officers/coordinators, policy developers, senior managers, program managers and directors), health-care professionals (dieticians and nurse practitioner) and government service employees (policy analyst and provincial program manager). | **Interviews with Syrian refugees**  **Tool:** Toolkit developed to review the interview guide, no other information provided  **Method**: Semi-structured interviews  **Details**: Conducted at resettlement service agency locations and community events in Arabic (based on preference)  **Facilitators**: Bilingual interviewers (English and Arabic) hired. Trained on the purpose of the project & ethical protocols  **Data collected**: The challenges, barriers, cultural and  gendered nature of FS and the adequacy of support services based on their experiences.  **Interviews with key informants**  **Method**: Semi-structured interviews  **Details**: Face-to-face interviews conducted at resettlement service agency locations and community events or via telephone, and in English (based on preference)  **Facilitators**: as above  **Data collected**: The capacity of service providers and agencies to support and respond to the FS issues facing Syrian  refugees. | Thematic analysis  **Transcription**: Interviews audio recorded, translated (as required) and transcribed.  **Analysis**: Use of NVivo to looking at patterns repeatedly to identify the intersectional nature of conceptual themes.  1. Open coding procedures to develop emerging data categories, key points, themes and patterns were recorded during iterative reviews of the data.  2. Data categories were then synthesized compared and assessed to capture different dimensions of meaning  3. Selective coding was conducted to integrate the categories into broader conceptual themes |
| **Mixed Methods Studies (n=69)** | | | | | | |
| Gichunge et al. (2016)^5^ | **Design:** Mixed Methods cross sectional sequential explanatory mixed methods  **Methodology:** Not stated  **Location:** South- East Queensland, Australia  **Dates:** April 2012 and April 2013 | To examine the association between household availability and consumption of traditional African vegetables among resettled African refugees residing in Southeast Queensland, Australia. | None stated Note: first author is native speaker of Swahili. | ***Quantitative*** | | |
|  |  |  |  | **Sample size**: 71 household  **Ethnicity**: Burundi 75%, Congolese and Rwandan **Immigration Status**: Not stated **Sex**: 89% Female, 11% male  **Time since resettlement**: Not stated **Inclusion/Exclusions**:  Primary food preparers from households with children under 18 yrs were recruited  **Recruitment:** Purposive recruited from African churches, community meetings and settlement agencies then snowballing | **Tool:** Questionnaire details not provided  **Method**: Interview **Details**: Interviews conducted in English or Swahili. Participants received AUD$25 grocery voucher. **Facilitators**: Researcher. No statement regarding training **Data collected**: demographics and socioeconomic characteristics, food environment and household food inventory using a pre-determined list of household food inventory | Chi-square; logistic regression |
|  |  |  |  | ***Qualitative*** | | |
|  |  |  |  | **Sample size**: 15  **Ethnicity**: Distribution not provided **Sex**: Distribution not provided **Time since resettlement**: Distribution not provided **Inclusion/Exclusions**: as above  **Recruitment:** Purposively selected from quantitative participants (no further details) | **Tool:** Three questions developed to further explore quantitative findings. Reviewed as part of a data collection and analysis cycle until saturation of information achieved  **Method**: Interview **Details**: Conducted in English or Swahili **Facilitators**: Not stated **Data collected**: Where do you get the vegetables? Why do you have these vegetables in your home? What problems do you encounter when sourcing your traditional vegetables in your neighbourhood? | Text analysis **Transcription**: Recordings of interviews transcribed verbatim  **Analysis**: Read several time to understand content. Line by line analysis of transcripts to identify categories |
| Hadley et al. (2007)^13^ | **Design:** Mixed Methods exploratory cross sectional (food security status collected 6 mths later) and exploratory ethnographic  **Methodology:** Ethnographic  **Location:** United States  **Dates:** Not stated | To use an exploratory ethnographic approach to examine FI and its manifestations among members of a resettled West African refugee community and then to assess the magnitude and severity of FI using a survey of West African refugees living in a mid-sized United States city. Secondary objectives were to examine the relationships between socio-economic status and indicators of acculturation and HFI | ***Quantitative*** | | | |
|  |  |  | Informed by qualitative work below (Informal participant observation in several health meetings, discussions with a nurse and social worker who were active in the community, informal interviews with refugees) | **Sample size**: 101 **Ethnicity**: Liberian **Immigration Status**: Not stated **Sex**: Female **Time since resettlement**: Avg 22.1 mths **Inclusion/Exclusions**: 18 yrs or older, has a child under 5 yrs of age, living in the United States for less than 4 yrs, and claimed Liberia as country of birth  **Recruitment:** Service-based convenience and snowballing sampling. Recruited through primary resettlement agency, meeting points of the Women, Infants, and Children Program (WIC; a public assistance programme with set income criteria), church groups | **Tool:** Survey. No information on piloting of survey, unclear if pre-tested  **Method**: Interviews **Details**: Conducted in English (national language of Liberia) at participants home **Facilitator**: West African women. Trained through role playing and several pre-tests (during pilot) **Data collected (baseline)**: Migration history, current household composition and economics, participation in food stamp programs, perceived difficulty with shopping and language. **Data collected (6mth follow up)**: HFI in previous 6 mths | Chi-square test; Wilcoxon two-sample; Spearman test; multivariate regression model. Measures of acculturation developed using PCA |
|  |  |  | ***Qualitative*** | | | |
|  |  |  | Ethnographic qualitative study to inform quantitative above | **Methods 1-3:** N/A  **Method 4 (formal interviews) Sample size**: 15 **Ethnicity**: Liberian **Sex**: Female **Time since resettlement**: <4yrs **Inclusion/exclusions**: 18 yrs or older, has a child under 5 yrs of age, living in the United States for less than 4 yrs, and claimed Liberia as country of birth  **Recruitment:** same as quantitative | **Method 1**: Observations **Details**: Patient observations in health meetings **Method 2**: Conversations **Details**: Discussions with nurse and social worker active in the community **Method 3**: Interviews (informal) **Details**: Informal with refugees  **Tool:** Topic guide **Method 4**: Interviews (formal) **Details**: Not stated **Facilitator**: West African women (see above) **Data Collected**: FI, dietary acculturation, food preparation and difficulties in the United States. | Not stated **Transcription**: Not stated **Analysis**: Key themes and illustrative quotes were collected |
| Henderson et al. (2017)^49^ | **Design:** Mixed methods (only qualitative relates) cross-sectional mixed methods  **Methodology:** Not stated  **Location:** Winnipeg, Canada  **Dates:** Not stated | To develop a greater understanding of the food and nutrition challenges facing newcomers in Winnipeg's North end, as well as identify any gaps in community resources and programming. | Conducted in partnership with 2 community based orgs: one engaged in food security and resettlement process. Researcher embedded in community prior to study (time period not specified). | **Sample size**: 12 (8 newcomers and 4 community workers) **Ethnicity**: Afghanistan, Bhutan, Burma, Congo, Iraq and the Philippines **Immigration Status**: 7 newcomers had refugee status (7/8) **Sex**: 2 men & 6 women **Time since resettlement**: 6mths - 6yrs **Inclusion/Exclusions**: Length of time in Canada ≥ 6mths, a predominant role in food procurement and preparation in the household, ≥18 yrs old and interest in participating in a photovoice study. Newcomers who had a family member participating in the study were excluded. 4 community workers were recruited who: were involved with relevant programmes for North End community members, including nutrition education, cooking classes and gardening programmes.  **Recruitment:** Purposively recruited through partner organizations, community workers and word of mouth | **Method**: Photovoice **Details**: Provided with single use camera and instructed to take 15 photos of food environment. Written instructions also provided. Cameras collected 6-14 days later by researcher **Data collected**: Photos of the food environment and experiences eg food purchases, food preparation and gardening activities  **Tool:** Semi-structured  **Method**: Interview **Details**: newcomers from the community and community workers involved in food and newcomer programming. Conducted in English with interpreters where required. Photographs bought to interview for discussion. Participants were paid CAD$25. **Facilitator**: Not stated. No statement regarding training **Data collected**: Photos and the food environment.  **Method**: Analytical memos **Details**: Recorded throughout the study to evaluate effectiveness of interview questions, and document aspects of interview not captured in transcriptions. | Thematic analysis **Transcription**: Interviews digitally recorded, transcribed by researcher and trained transcriber **Analysis**: Started during interviews. Transcriptions analysed for emerging themes (first level codes) then inductive codes with field notes. Comparison between codes to develop hierarchy (second level codes). This developed detailed understanding of the food-related issues facing newcomers |
| Judelsohn et al. (2017)^50^ | **Design:** Mixed methods (only qualitative relates) cross-sectional  **Methodology:**  Participatory action research  **Location:** Buffalo, NY, United States  **Dates:** Not stated | To explore the experiences of refugees from Burma in navigating food environments in the United States, and explore the extent to which local governments are supporting or hindering their access to culturally preferred, nutritious foods. | Transdisciplinary research project, Dealing with Disparities in Food Access Among (Burmese) Refugees (DDFAR) appointed a Community Advisory Group (CAG), comprising 5 members of the refugee community and two representatives of organizations that serve the Burmese refugees from Burma in Buffalo, to guide the research | **Sample size**: 28 **Ethnicity**: Burmese **Immigration Status**: Refugee status **Sex**: 79% female 21% male **Time since resettlement**: >6mths **Inclusion/Exclusions**: Born in Burma, refugee status, lived in United States > 6mths, ≥ 18 yrs old  **Recruitment:** Snowballing recruited by CAG members and research team members (ties to Burmese community) through word of mouth and fliers | **Tool:** Open ended semi-structured tested in local communities to check cultural appropriateness and fidelity of instrument.  **Method**: Interview **Details**: conducted in preferred language, Burmese, Karen or English at preferred location. **Facilitator**: Bilingual with ties to Burmese and Karen communities. Accompanied by additional research team member. No statement regarding training. **Data collected**: How refugees navigate the food environment, challenges and how they overcome these.  **Other methods (not applicable for SR)**: Interviews with local govt (n=7) and civil society representatives (n=6), review of policy documents and United States Census Bureau data | Not stated **Transcription**: Interviewers transcribed into English **Analysis**: Not stated |
| Nunnery and Dharod (2017)^51^ | **Design:** Mixed methods (only qualitative relates) cross-sectional case study  **Methodology:** Not stated, analysed by pre-and post-resettlement factors  **Location:** County in South-eastern United States  **Dates:** March 2010 to November 2012 | 1. To examine the socio-demographic characteristics and prevalence of FI in three groups of refugees resettled in the United States;  2. To describe themes that arose as potential determinants of FI for refugees; and  3. To posit a conceptual model of the potential determinants of FI for refugees and how they interrelate. | Community health workers living in the community of same ethnicity conducted recruitment and interviews.  Findings shared with community interviewers to validate findings and provided their explanations and perceptions into the findings. | **Sample size**: 97 (secondary data - combination of 3 studies all using same protocol) **Ethnicity**: Liberian (n=33), Sudanese (n=22) Montagnard’s (n=42) **Immigration Status**: Refugee status or family reunification **Sex**: Women **Time since resettlement**: Avg of 8yrs **Inclusion/Exclusions**:  1. Came to the United States under refugee status or under the family reunification program 2. 18 yrs of age or older  3. the main meal preparer of their household  4. Had children younger than 18 yrs of age  **Recruitment:** Snowball techniques such as networking, telephone invitations and referrals. | **Tool:** Semi-structured questionnaire - no further details provided on how created  **Method**: Secondary data analysis of 3 studies (Interviews collecting qualitative and quantitative data) **Details**: Conducted in participants home. Liberian interviews in English, Sudanese and Montagnard in native language **Facilitators**: Community health workers of same ethnicity and living in the community of study group in presence of research team member. Bilingual in English and native language. Trained to conduct interviews and follow protocol. **Data collected**:  *Quantitative*: sociodemographic & FS  *Qualitative*: 1) general experiences related to social and cultural changes during initial period of resettlement; 2) current lifestyle, food shopping and dietary habits; 3) pre-resettlement living conditions, health and food environment; 4) concerns and issues related to food and health in the United States. | Not stated **Transcription**: Interviews audio recorded except Sudanese interviews (not culturally appropriate) and transcribed verbatim. Observational field notes also used in data analysis. **Analysis**:  Data organised under two main themes: 1. differences in current and past living conditions and 2. differences in current and past food choices. Steps included reviewing, comparing, labelling and categorising data into common themes. Repeated observations in data coded as major themes. 2 researchers: independent categorisation then comparison between researchers. |
| Peterman et al. (2013)^17^ | **Design:** Mixed methods cross sectional, methodology not stated  **Location:** Lowell, Massachusetts, United States  **Dates:** Survey - September to November 2007 and April to June 2008  Focus Groups - April to May 2007 | To examine whether high rates of FI persist among women in a well-established refugee population, to consider which characteristics are associated with FI | ***Quantitative*** | | | |
|  |  |  | Conducted in conjunction with Cambodian Community Health (CCH) 2010, the Cambodian Mutual Assistance Association of Greater Lowell (CMAA, CCH 2010 partner) and the Lowell Community Health Center (CCH 2010 lead agency) conducted a study of past and current food experiences of Cambodian refugee women. | **Sample size**: 150 (data analysis) of 160 completed surveys of 196 households selected **Ethnicity**: Cambodian **Immigration Status**: Not stated **Sex**: Female **Time since resettlement**: Avg of 19.3 yrs in United States **Inclusion/Exclusions**: Cambodian women aged between 35 to 60 yrs  Analysis conducted only on those that had been in the United States for ≥ 5 yrs  **Recruitment:** Random sample selected from 2007 Lowell City Census and telephone book; registered voters from University of Massachusetts Lowell; and clients from community agencies. | **Tool:** Survey developed out of focus groups (qualitative section of study) and translated and blind-back translated.  **Method**: Survey  **Details**: Administered in person in preferred language of participant at participants house  **Facilitators**: Survey administrators. Trained, but details not provided **Data collected**: HFS, depression, acculturation, food stamp participation, and demographics | Logistic regression |
|  |  |  | ***Qualitative*** | | | |
|  |  |  | See above | **Sample size**: 11 (2 focus groups) **Ethnicity**: Cambodian **Immigration Status**: Not stated **Sex**: Female **Time since resettlement**: Not stated **Inclusion/Exclusions**: Women aged 30-65yrs  **Recruitment:** Recruited from clients of CMAA | **Tool:** Semi-structured moderator guides  **Method**: Focus Groups (n=2) **Details**: Conducted in Cambodian, no other details provided **Facilitators**: English speaking moderator (translated). No statement regarding training **Data collected**: participants’ experiences with food on arrival in the United States and current access to food. | Directed content analysis **Transcription**: Focus groups take recorded and transcribed in English **Analysis**: Not stated |

Legend: Avg = Average, Yrs = Years, Mths = Months
